# Ventilatory efficiency during constant-load test at lactate threshold intensity: Endurance versus resistance exercises

**DOI:** 10.1371/journal.pone.0216824

**Published:** 2019-05-21

**Authors:** Lluis Albesa-Albiol, Noemí Serra-Payá, María Ana Garnacho-Castaño, Lluis Guirao Cano, Eulogio Pleguezuelos Cobo, José Luis Maté-Muñoz, Manuel V. Garnacho-Castaño

**Affiliations:** 1 GRI-AFIRS, School of Health Sciences, TecnoCampus-Pompeu Fabra University, Mataró, Barcelona, Spain; 2 Department of Rehabilitation, Hospital Asepeyo, Sant Cugat, Barcelona, Spain; 3 Department of Physical and Rehabilitation Medicine, Hospital de Mataró, Mataró, Barcelona, Spain; 4 Department of Physical Activity and Sports Science, Alfonso X El Sabio University, Villanueva de la Cañada, Madrid, Spain; James Cook University College of Healthcare Sciences, BRAZIL

## Abstract

There is a lack of evidence about the ventilatory efficiency in resistance exercises despite the key role played in endurance exercises. This study aimed to compare the cardiorespiratory, metabolic responses and ventilatory efficiency between half-squat (HS) and cycle ergometer exercises during a constant-load test at the lactate threshold (LT) intensity. Eighteen healthy male participants were randomly assigned in a crossover design to carry out HS or cycle ergometer tests. For the three HS tests, a one repetition maximum (1RM) test was performed first to determine the load (kg) corresponding to the 1RM percentages. In the second test, the incremental HS exercise was carried out to establish the load (kg) at the LT intensity. Finally, a constant-load HS test was performed at the LT intensity. The first cycle ergometer test was incremental loading to determine the intensity in watts corresponding to the LT, followed by a constant-load test at the LT intensity. A recovery time of 48 hours between each test was established. During both constant-load test, cardiorespiratory and metabolic responses were monitored. A significant exercise mode x time interaction effect was only detected in oxygen uptake (VO_2_), heart rate, and blood lactate (p < 0.001). No differences were found between the two types of exercise in ventilatory efficiency (p >0.05). Ventilation (VE) and carbon dioxide were highly correlated (p <0.001) in the cycle ergometer (r = 0.892) and HS (r = 0.915) exercises. In the VO_2_ efficiency slope (OUES), similarly significant and high correlations (p <0.001) were found between VO_2_ and log_10_ VE in the cycle ergometer (r = 0.875) and in the HS (r = 0.853) exercise. Although the cardioventilatory responses were greater in the cycle ergometer test as compared to HS exercise, ventilatory efficiency was very similar between the two exercise modalities in a predominantly aerobic metabolism.

## Introduction

Recent studies have used the lactate threshold (LT) or the ventilatory threshold as parameters to monitor and assess cardiorespiratory responses [1, 2, 3], slow component of oxygen uptake, and gross mechanical efficiency [4] in unusual resistance exercises using a cardiopulmonary exercise tests (CPET), as also occurs in endurance exercise [5–9]. During constant-load test at a load intensity equivalent to the LT, it was observed a greater cardiorespiratory response to cycle ergometer exercise compared to the half-squat (HS). The cardiorespiratory and metabolic response was stable in both types of exercise; greater muscular fatigue was observed after completion of the HS test [2]. As could be expected, resistance exercises increased local muscular fatigue in the lower limbs, while endurance exercises increased cardiorespiratory response.

Cardiorespiratory fitness is frequently evaluated by means of ventilatory efficiency [10, 11]. The fundamental cause of ventilatory efficiency is the matching of ventilation (VE) and perfusion in the lungs. The mismatching of perfusion and VE diminishes the efficiency of lung gas exchange, demanding an increase in VE for a given CO_2_ output and arterial PCO_2_. This mismatching phenomenon contributes essentially to hyperpnea and dyspnea [12] affecting ventilatory performance. It is common to assess ventilatory efficiency in endurance exercises mostly in different types of diseases or pathologies [13–14], in sports performance [11], and in healthy subjects [10, 15], establishing the slope of the linear relationship between VE and carbon dioxide (VE/VCO_2_ slope) during an incremental test up to the anaerobic [16] or ventilatory threshold [17] and the ventilatory compensation point [10]. Another option to quantify ventilatory efficiency in endurance exercises is to determine the oxygen uptake efficiency slope (OUES). The OUES indicates how effectively oxygen is extracted and taken into the body during incremental exercise [17]. The OUES is considered a very appropriate tool in the evaluation of cardiovascular fitness in overweight adolescents [18], the severity of heart disease [19], the effects of physical training or treatment [20, 21], and the risk of a serious or fatal event [22].

Although many studies have analyzed the slope of VE/VCO_2_ and OUES by age, sex, fitness level, and diseases in endurance exercises [10, 11, 14, 17, 19], it is unusual to observe studies comparing VE/VCO_2_ slope and OUES between different exercise modalities [23, 24]. Sun et al. [10] demonstrated that VE/VCO_2_ slope is not exercise mode-dependent, however, Davis et al. detected that VE/VCO_2_ slope was lower on the cycle ergometer than the treadmill in women but not in men [25]. For OUES, treadmill values were higher than cycle ergometer [24]. Recently, Salazar-Martinez et al. demonstrated that ventilatory efficiency was unaffected by ergometer type [26]. The assumption that ventilatory efficiency could be similar between different exercise modalities is controversial and more research is needed to compare several exercise modes. Despite the importance that has been given in the scientific literature to the assessment of ventilatory efficiency in endurance exercises in healthy people and especially in the clinical settings, it is a field of knowledge that needs to be explored in resistance exercises. There are no previous data regarding VE/VCO_2_ slope and OUES in HS exercise and, to the best of our knowledge, ventilatory efficiency has not been compared between resistance and endurance exercises.

In cardiorespiratory fitness assessment, this knowledge could have an added value in selecting the type of exercise to improve ventilation efficiency. If resistance exercises demonstrate adequate ventilatory efficiency, professionals in the health field could use resistance training to increase local muscle endurance while maintaining good ventilatory efficiency.

It is common to assess ventilatory efficiency during incremental endurance tests, however, prolonged constant-load endurance tests can be recommended as a good option in the clinical health setting to determine the VE/VCO_2_ slope [27, 28] or OUES because they do not subject the participants to significant cardiorespiratory, metabolic, and muscular stress. A constant-load test at LT intensity might be an interesting alternative for applying to healthy people in both endurance and resistance exercises to assess ventilatory efficiency without inducing a strenuous cardiorespiratory and metabolic stress.

The main objective of this study was to compare the ventilatory efficiency, measured by OUES and VE/VCO_2_ slope, and cardioventilatory responses of HS and cycle ergometer exercise in a constant-load test at LT intensity. A secondary goal was to determine the relationship between the OUES and the VE/VCO_2_ slope in both exercise modalities in each participant.

## Material and methods

### Participants

Eighteen healthy male participants were recruited among the students of the Department of Physical Activity and Sports Sciences (age: 21.8 ± 1.5 years, height: 180.3 ± 5.7 cm, weight: 82.6 ± 9.0 kg, body mass index: 25.4 ± 2.0). All participants had at least 6 months of resistance training experience and were completely familiar with the HS exercise and the cycle ergometer.

Four exclusion criteria were established: 1) any cardiovascular, metabolic, neurological, pulmonary, or orthopedic disorder that could limit exercise performance, 2) the use of any medication, supplements, or substance that could improve performance, 3) 1RM ≤150 kg in the exercise of the HS, 4) elite athlete status.

Eligible participants were informed of the tests to be performed and those who agreed with the study protocols signed their written consent to participate. The subjects were instructed to abstain from other exercise or training during the two-week study period. The study protocol adhered to the principles of the Declaration of Helsinki for studies with human beings and was approved by the Ethics Committee of the Alfonso X El Sabio University (Villanueva de la Cañada, Madrid, Spain).

### Experimental design

The participants visited the Exercise Physiology Laboratory five times during the two-week study period, at the same time of day (± 2 hours) and in similar environmental conditions (room temperature 21–25°C, atmospheric pressure 715–730 mm Hg, relative humidity ~ 45%). Participants were randomly assigned in a crossover design to perform HS or cycle ergometer tests. A rest period of 48 hours was established between each of the five tests. The protocols were implemented according to procedures previously established by our research group [2].

For the three HS tests, a one repetition maximum (1RM) test was performed first to determine the load (kg) corresponding to the 1RM percentages to be used during the second test, the incremental HS exercise to establish the load (kg) at the intensity corresponding to the LT. Finally, a constant-load HS test was performed at the LT intensity established during the incremental exercise test. The first cycle ergometer test was incremental loading to determine the intensity in watts (W) corresponding to the LT, followed by a constant load test at the LT intensity. During both constant-load test, acute cardiorespiratory and metabolic responses were monitored. The timing of the blood lactate sampling was the same for both the HS and cycle ergometer testing.

### Half squat tests

In the HS tests, a Smith machine (Matrix Fitness, Johnson Health Tech, Cottage Grove, MN, USA) was used to ensure safe and controlled movements. HS technique was determined as in previous studies [3, 29]. The variation in range of motion (ROM) during HS exercise was accurately determined during a familiarization session and in all the tests. Participants positioned themselves under the barbell in an upright position with the knees and hips fully extended and legs spread approximately at the shoulders’ width. The barbell was placed on the upper back (trapezius muscle), approximately at the level of the acromion. During the descent of the bar, participants flexed the knees and hips (eccentric action) to lower the barbell in a controlled manner, until 90^0^ flexion of the knees [30]. From this position, the concentric muscle action was started until fully extending the knees and hips. The body position was individually adjusted and exactly replicated on each HS test.

Each HS test started with a 5-minute low-intensity general run and 5-minute general joint mobility warm-up, followed by a specific warm-up of a series of 3–5 repetitions (HS) at a relative intensity of 40–60% of 1RM.

#### 1RM test

After a 2-minute rest, the HS test protocols began. To determine 1RM, 3–5 series were carried out, using an increasing weight each time. The 1RM was defined as the last load lifted by the subject, completing a knee extension to the required position. The rest period between each attempt was 4 minutes.

#### Incremental HS test

The incremental HS test was carried out in 6 one-minute series, at relative intensities of 10%, 20%, 25%, 30%, 35%, and 40% 1RM as described in previous studies [2, 4, 29]. In each series, 30 repetitions of 2 seconds each were performed (1 second for eccentric muscle action and 1 second for concentric action), using a metronome to establish the rhythm; a member of the research team provided visual and verbal cues to maintain an adequate rate. A passive rest period of 2 minutes between series was established. During this period, blood samples were collected by an experienced researcher and the corresponding load was increased. The test ended when the repetitions were no longer executed correctly or was voluntarily terminated by the participant when he could not perform the repetitions at the established cadence. Blood samples (5 μL) were obtained by pricking the finger 30 seconds after the end of each series. Lactate levels were measured using a portable analyzer (Lactate Pro LT-1710, Arkray Factory Inc., KDK Corporation, Siga, Japan).

Based on the algorithmic adjustment method described by Orr et al. [31], LT was defined as the load intensity at which blood lactate concentrations begin to increase exponentially [32]. LT was detected by two-segment linear regression, placing the 2 emergent linear regression equations for each segment at the point of intersection between a plot of blood lactate concentration and relative intensity [33]. Data analysis was done using Matlab version 7.4 (MathWorks, Natick, MA, USA).

#### Constant-load HS test

In the constant load HS test, 21 sets of 15 repetitions were performed. The duration of each set was 30 seconds (1 second each for the eccentric and concentric phases, guided by a metronome and visual and verbal signals), with 1-minute rest between sets. The entire constant-load test lasted 31 minutes. Respiratory exchange data were recorded during the constant-load test using a breath-by-breath open-loop gas analyzer (Vmax spectra 29, Sensormedics Corp., Yorba Linda, California, USA), previously calibrated. VO_2_, VE, VCO_2_, and respiratory exchange ratio (RER) were monitored. The heart rate was quantified every 5 seconds by telemetry (RS-800CX, Polar Electro OY, Finland). To determine lactate concentrations, finger-prick blood samples were obtained, as described for the incremental test, at rest and 30 seconds after the end of 7 HS sets (S): S3, S6, S9, S12, S15, S18 and S21.

### Cycle ergometer tests

The incremental and constant-load tests on a cycle ergometer (Monark ergomedic 828E, Vansbro, Sweden) included a 5-minute warm-up at a pedaling rate of 50 rev.min^-1^ and a load of 50 W, followed by 5 minutes of dynamic joint mobility and stretching exercises. The load during the incremental and constant-load tests was defined according to the characteristics of the cycle ergometer, as previously described [34]. Briefly, pedaling at an intensity of 50 W is the same as pedaling at a rate of 50 rev.min^-1^ at a load of 1-kilogram force (kgf). To increase the load by 25 W during an incremental protocol, pedaling cadence at a rate of 50 rev.min^-1^ should be performed at a load equivalent to 0.5 kfg. After 2 minutes of rest, the specific tests on the cycle ergometer began.

#### Incremental cycle ergometer test

The incremental test using a ramp protocol that started with a load of 50 W (50 rev.min^-1^ at a load of 1kgf), increased in steps of 25 W.min^-1^ until completion of 8 min at a pedaling rate of 50 rev.min^-1^ at a load of 0.5 kgf. Blood samples (5 μL) were obtained by finger pricking at rest and every 2 minutes during the incremental test. The LT was detected by inspecting the plot of blood lactate concentrations against the workload, according to the protocol described by Weltman et al. [35]. LT was defined as the highest exercise load completed when an increase of 0.5 mmol.L^-1^ was detected over baseline concentrations in at least 2 consecutive samples.

#### Constant-load cycle ergometer test

The constant load cycle ergometer test was performed with continuous pedaling at a rate between 70–80 rev.min^-1^ at an intensity (W) equivalent to the LT, previously determined in the incremental test. The load in kfg was individually adjusted to each subject at 70–80 rev.min^-1^ to develop the W corresponding to the LT intensity. Total duration of the test was 31 minutes. The blood lactate samples were obtained with the same portable analyzer as in the HS test, at the beginning of the test and (coinciding with the timing in the HS test) at the following minutes (M) thereafter: M4, M8.5, M13, M17.5, M22, M26.5, M31. During the constant-load test, respiratory exchange and heart rate data were recorded as described in the HS constant-load test.

### Ventilatory efficiency

The ventilatory efficiency of each participant was determined in two ways: 1) the slope of the relationship between VE and VCO_2_ during each constant-load test; 2) the OUES slope, calculated as the relationship between VO_2_ and the logarithm of the VE during the constant-load test: VO_2_ = a log10 VE + b).

### Statistical analysis

The Shapiro-Wilk test was used to verify the normal distribution of the data, reported as mean, standard deviation (SD), and confidence intervals (95% CI). To identify significant differences between the HS and cycle ergometer exercises in the cardioventilatory and lactate variables, a general linear model was performed with a two-way analysis of variance (ANOVA) for repeated measurements. The two factors were the exercise mode (HS or cycle ergometer) and time point (corresponding to 7 control points in both exercise modes). When appropriate, a post-hoc Bonferroni adjustment was implemented for multiple comparisons. To determine the differences between the two exercise modes in the VE/VCO_2_ and OUES slopes, Student-t was applied for related samples. The slope of VE/VCO_2_ and OUES was calculated by linear regression between VE and VCO_2_ and between VO_2_ and log10 VE, respectively. The Pearson product-moment correlation coefficients were calculated to determine significant relationships between the VE and the VCO_2_ and between the VO_2_ and the log10 VE, and to establish the possible relationship between the OUES and the VE/VCO_2_ slope.

Partial eta square (η_*p*_^2^) was calculated to determine the magnitude of the response in ANOVA analysis. Cohen´s *d* for the planned comparisons was used to determine effect sizes. A large effect size was defined as η_*p*_^2^ ≥ 0.26, *d* ≥ 0.80; moderate η_*p*_^2^ ≥ 0.13, *d* ≥ 0.40; and small η_*p*_^2^ < 0.02, *d* < 0.40 [36]. Statistical power (SP) was also determined. The intraclass correlation coefficients and the percentage of variation coefficients were calculated to determine the relative and absolute reliability. The level of significance was set at p <0.05. All statistical methods were performed using the SPSS Statistics software package version 23.0 for Macintosh (SPSS, Chicago, IL, USA). The graphics were made in the Microsoft Excel version 16.20 for Mac.

## Results

Anthropometric characteristics and incremental test data for the HS and cycle ergometer exercises are shown in Table 1.

**Table 1:** Descriptive data related to anthropometric characteristics, 1RM- and incremental-load tests.

Variables	Mean (SD)
**Participants**	N = 18
**Age (years)**	21.2 (1.5)
**Height (cm)**	180.3 (5.7)
**Weight (kg)**	82.6 (9.0)
**BMI (kg.m**^**-2**^**)**	25.4 (2.1)
**1RM in HS (kg)**	206.3 (36.4)
**HS load at LT (kg)**	51.2 (9.0)
**HS relative intensity at LT (%)**	25.8 (4.6)
**CYC load at LT (W)**	130.8 (24.8)

Abbreviations: 1RM = one-repetition maximum; BMI: body mass index; CYC: cycle-ergometer; HS = half-squat; LT = lactate threshold; SD = standard deviation.

Differences in cardioventilatory and lactate responses are shown in Table 2. The mean of the intraclass correlation coefficients and the coefficients of variation for cardioventilatory variables and lactate were 0.970 (0.942–0.987) and 6.7% ± 3.4%, respectively.

**Table 2:** Differences in cardioventilatory and lactate responses between half-squat vs cycle-ergometer during constant-load test at lactate threshold intensity.

	CYC (95% CI)	HS (95% CI)	*P*^*1*^ ES/SP	*P*^*2*^ ES/SP	*P*^*3*^ ES/SP
**VO**_**2**_ **(L.min**^**-1**^**)**	2.2	1.6	0.007	< 0.001	< 0.001
	(2.0–2.5)	(1.5–1.7)	0.2/0.9	0.6/1.0	0.6/1.0
**VO**_**2**_ **(mL.kg**^**-1**^**.min**^**-1**^**)**	19.8	27.8	0.517	< 0.001	< 0.001
	(18.6–20.9)	(24.8–30.8)	(0.1–0.3)	(0.2–1.0)	(0.6–1.0)
**VCO**_**2**_ **(L.min**^**-1**^**)**	2.1	1.5	0.062	< 0.001	< 0.001
	(1.8–2.3)	(1.4–1.6)	0.1/0.7	0.6/1.0	0.6/1.0
**VE (L.min**^**-1**^**)**	53.7	43.1	0.510	< 0.001	0.002
	(48.2–59.2)	(40.1–46.1)	0.1/0.3	0.4/1.0	0.5/0.9
**RER**	0.9	0.9	0.923	< 0.001	0.084
	(0.9–0.9)	(0.9–1.0)	0.0/0.1	0.5/1.0	0.2/0.4
**HR (beat.min**^**-1**^**)**	139.6	123.8	< 0.001	< 0.001	< 0.001
	(131.2–148.0)	(116.7–130.8)	0.3/1.0	0.6/1.0	0.6/1.0
**Lactate (mmol.L**^**-1**^**)**	2.6	2.8	< 0.001	< 0.001	0.148
	(2.2–2.9)	(2.6–3.1)	0.3/1.0	0.8/1.0	0.1/0.3

Abbreviations used: CYC: cycle-ergometer; ES: effect size; HR: heart rate; HS: half-squat; L: liter; min: minute; RER: respiratory exchange ratio; SP: statistical power; VCO_2_: carbon dioxide production; VE: minute ventilation; VO_2_: oxygen uptake. *P*^*1*^ Significant differences for exercise mode x time interaction effect. *P*^*2*^ Significant differences for time effect. *P*^*3*^ Significant differences for exercise mode effect. Data are provided as mean and 95% confidence intervals (95% CI).

**Fig 1:**
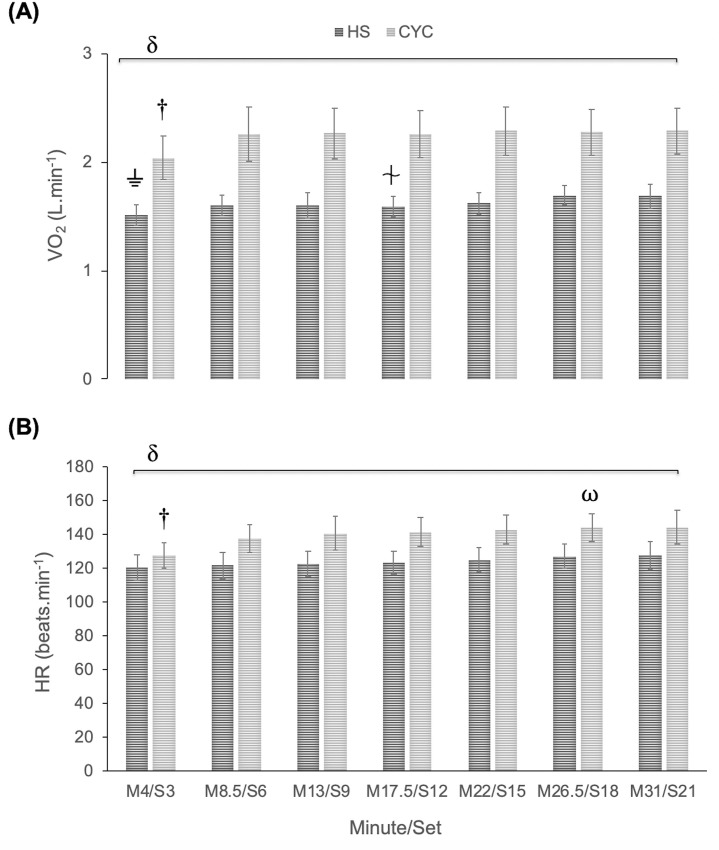
**Multiple comparisons between cycle ergometer (CYC) and half-squat (HS): (A) Oxygen uptake (VO**_**2**_**). (B) Heart rate (HR).** δ Significant differences p < 0.05 between cycle ergometer and half-squat at each checkpoint. † Significantly different from M8.5, M13, M17.5, M22, M26.5, M31 in cycle ergometer, p < 0.01. ⍵ Significantly different from M8.5, M17.5 in cycle ergometer, p = 0.017. ⏚ Significantly different from S6, S18, S21 in HS exercise, p < 0.05. ⏆ Significantly different from S21 in HS exercise, p = 0.026.

For absolute VO_2_, a significant exercise mode x time interaction effect was observed (p = 0.007, F_(6, 102)_ = 3.18). Bonferroni test confirmed that VO_2_ was significantly higher in cycle ergometer than HS exercise at the 7 established control points (p <0.001; large effect *d* ≥ 1.64). In cycle ergometer, a significant lower VO_2_ was detected in M4 regarding the rest of the control points (p ≤ 0.002; moderate effect *d* ≥ 0.46 and ≤ 0.60). However, a VO_2_ stabilization was observed after M8.5 (p > 0.05). In HS exercise, a significant increase in VO_2_ was observed (p < 0.05) in S3 with respect to S6 (moderate effect, *d =* 0.46), S18 (large effect, *d =* 0.95), and S21 (large effect, *d =* 0.86) (Fig 1A).

No significant exercise mode x time interaction effect was found for the relative VO_2_ (p > 0.05) and VE variable (p > 0.05).

For heart rate, a significant effect (p <0.001) was observed for exercise mode x time interaction (F_(6, 102)_ = 5.85). The Bonferroni test determined that heart rate was significantly lower in HS exercise than in cycle ergometer test at the 7 established control points (p <0.05; in M4/S3, moderate effect *d =* 0.49; rest of control points large effect *d* ≥ 0.94). In cycle ergometer exercise, a significant increase in heart rate was confirmed in M4 regarding all control points (p < 0.01; moderate effect versus M8.5 and M13, *d* ≥ 0.62 and ≤ 0.74; large effect versus M17.5, M22, M26.5, M31, *d* ≥ 0.83) (Fig 1B).

**Fig 2:**
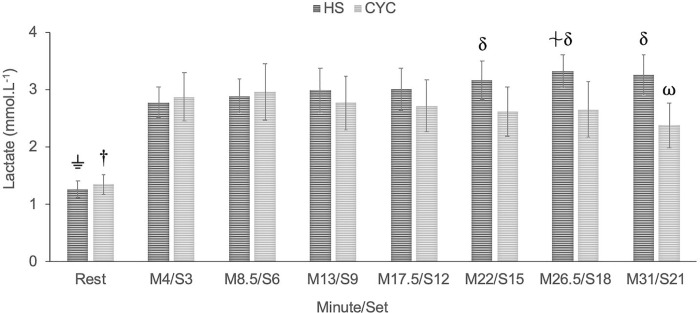
Multiple comparisons between cycle ergometer (CYC) and half-squat (HS) in blood lactate. ⏚ Significantly different from S3, S6, S9, S12, S15, S18, S21 in HS exercise, p < 0.001. † Significantly different from M4, M8.5, M13, M17.5, M22, M26.5, M31 in cycle ergometer, p < 0.01. δ Significantly different from cycle ergometer in M22/S15, M26.5/S18, M31/S21, p < 0.05. ⍵ Significantly different from M4 in cycle ergometer, p = 0.028. ⏆ Significantly different from S3 and S6 in HS exercise, p < 0.05.

Blood lactate concentrations indicated a significant exercise mode x time interaction (p < 0.001, F_(7, 119)_ = 6.93). The Bonferroni adjustments showed a significant increase from rest period in both exercise modes (p ≤0.005; large effect *d* ≥ 1.71). Significant higher blood lactate levels were found in HS exercise regarding cycle ergometer (p <0.05) at control points M22/S15 (moderate effect, *d* = 0.72), M26.5/S18 (large effect, *d* = 0.82), and M31/S21 (large effect, *d* = 1.19) (Fig 2).

In the RER, no significant interaction effect was observed for exercise mode x time (p > 0.05).

**Fig 3:**
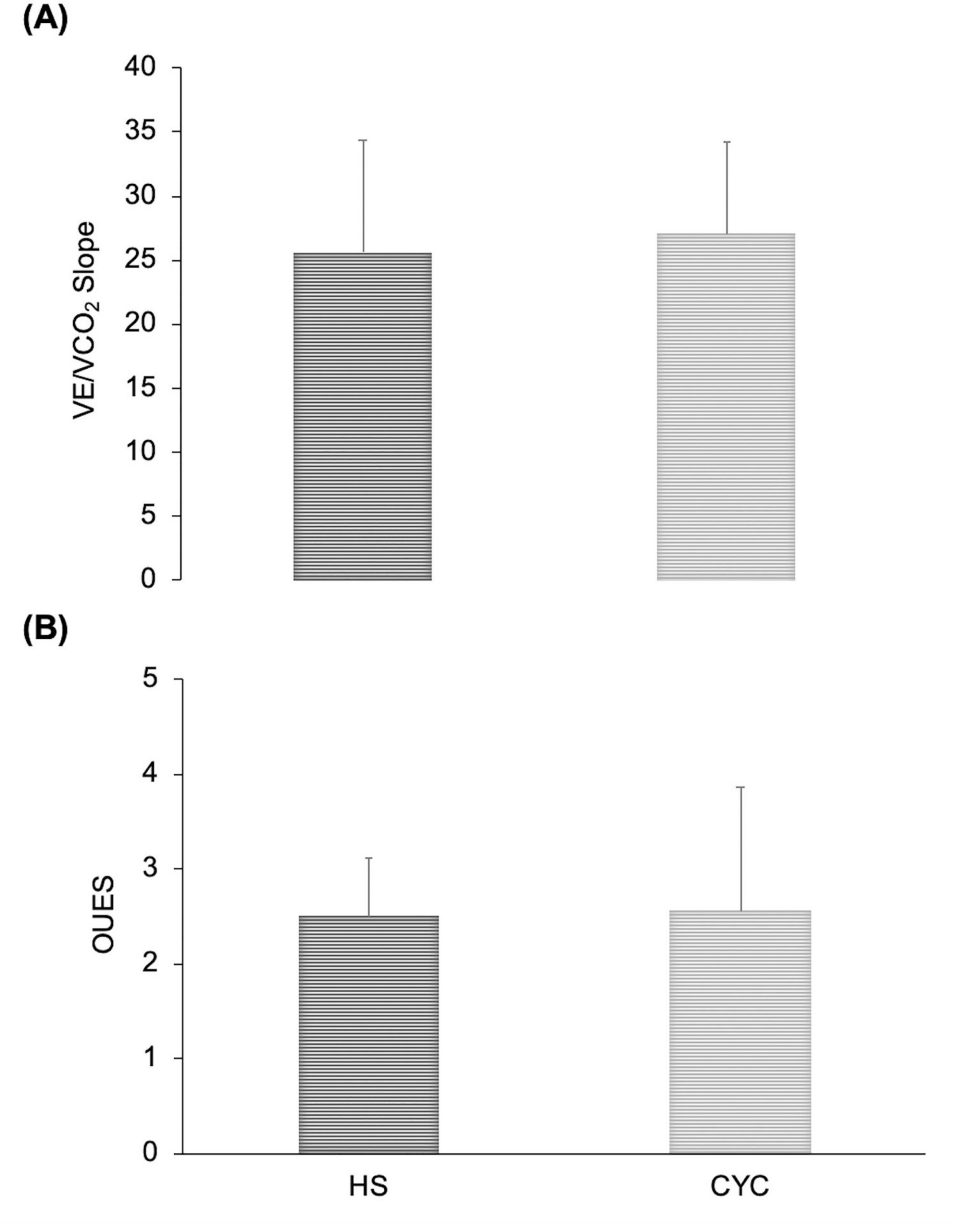
Differences between cycle ergometer (CYC) and half-squat (HS) in the VE/VCO_2_ slope and OUES. No significant differences between both exercise modalities.

Regarding VE/VCO_2_ slope and OUES, no differences were found between the two types of exercise (p >0.05) (Fig 3).

**Fig 4:**
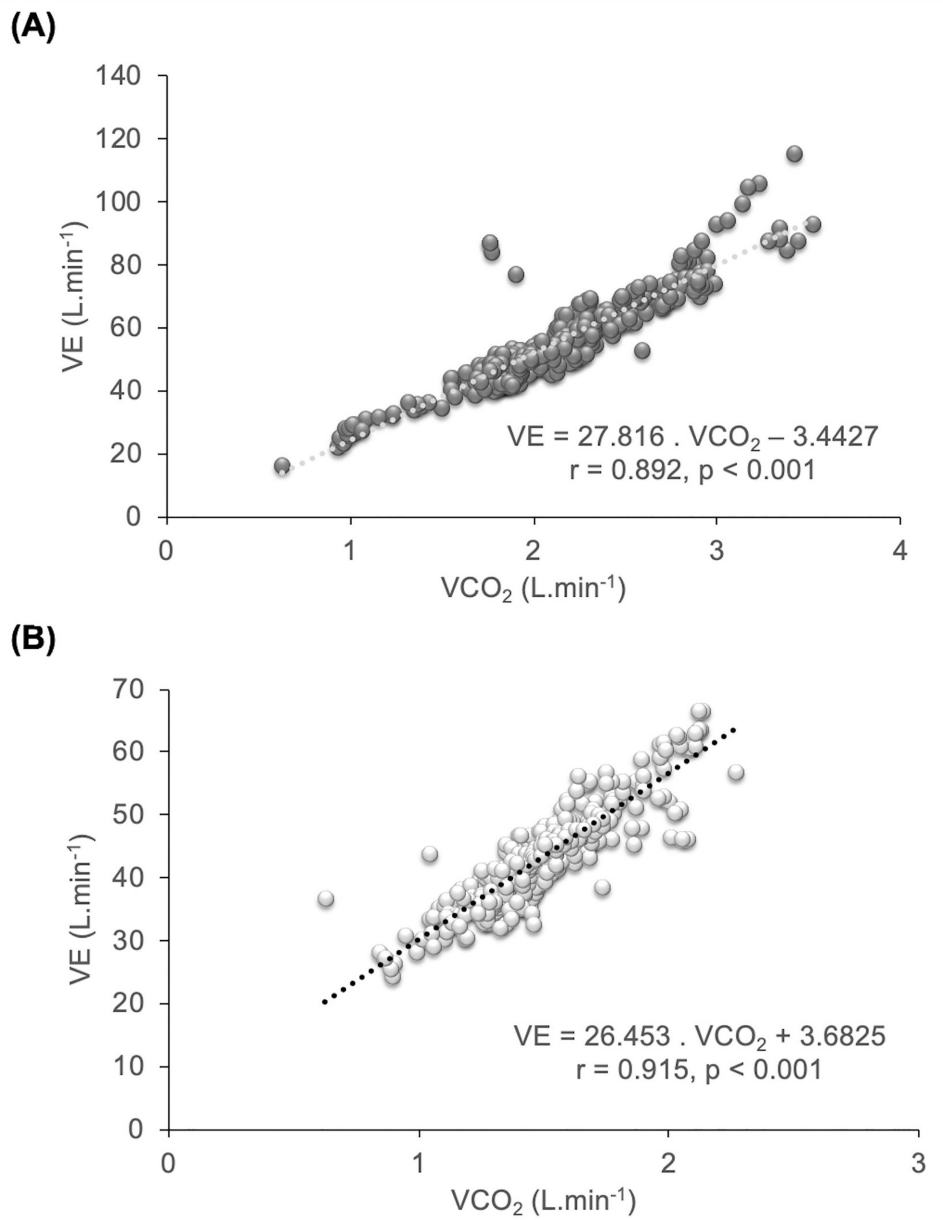
Linear relationship between ventilation (VE) and carbon dioxide (VE/VCO2 slope): (A) Cycle ergometer (CYC). (B) Half-squat (HS).

In the VE/VCO_2_ slope, VE and VCO_2_ were highly correlated (p <0.001), both in the cycle ergometer (r = 0.892) and HS (r = 0.915) modalities (Fig 4A and 4B, respectively).

**Fig 5:**
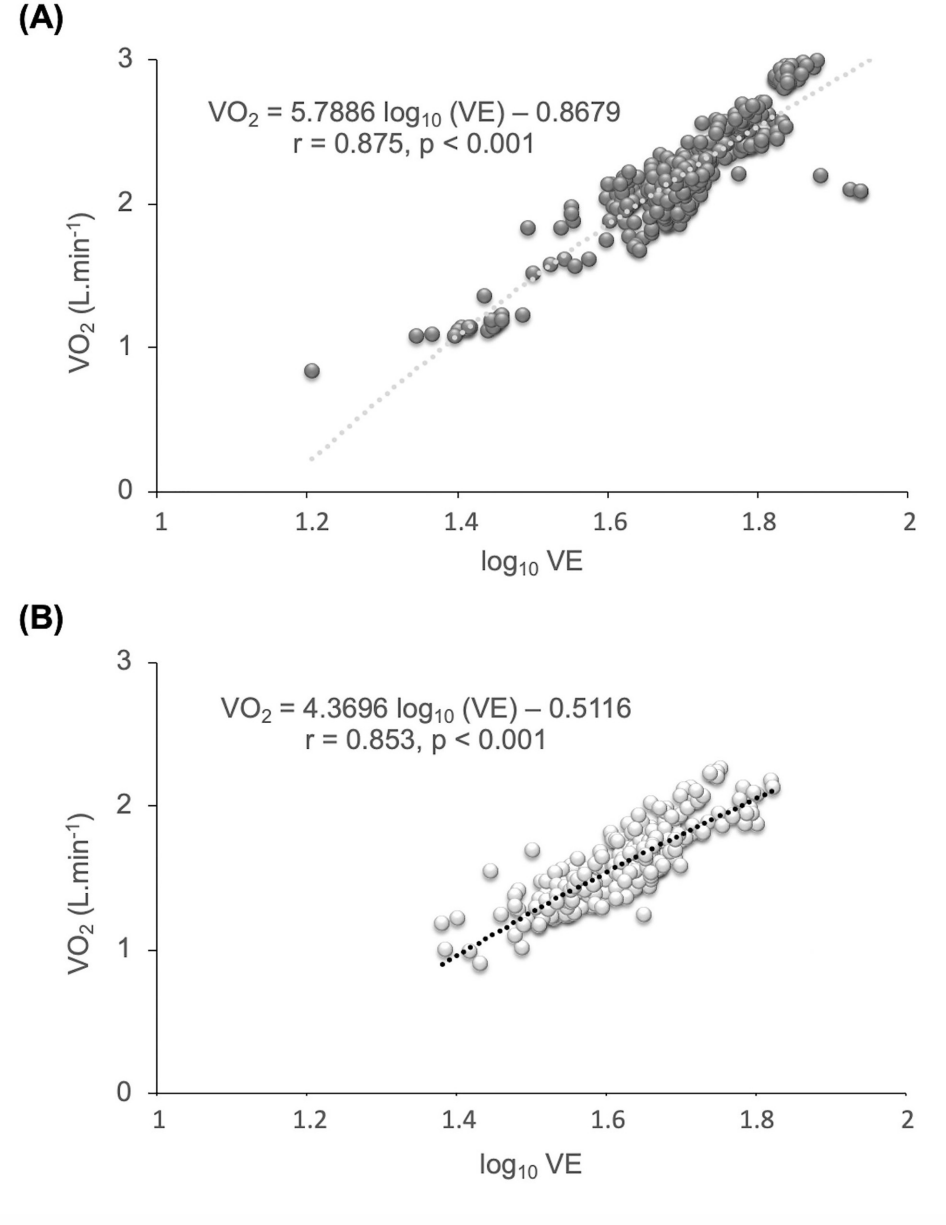
Relationship between oxygen uptake (VO_2_) and log_10_ VE (OUES): (A) Cycle ergometer (CYC). (B) Half-squat (HS).

In the OUES, similarly high correlations (p <0.001) were found between VO_2_ and log_10_ VE in the cycle ergometer (r = 0.875) and in the HS (r = 0.853) (Fig 5A and 5B, respectively).

No significant correlation was found between the OUES and the slope of the VE/VCO_2_ in the HS (r = -0.345, p = 0.160) nor in the cycle ergometer (r = 0.315, p = 0.203). Also, no significant correlation was observed between the HS and cycle ergometer modes in OUES (r = 0.356, p = 0.147) or VE/VCO_2_ slope (r = 0.422, p = 0.081).

## Discussion

To the best of our knowledge, this study applied two novelties in methodological approach, with respect to previous research. First, it determined ventilatory efficiency in HS exercise by two distinct methods (VE/VCO_2_ slope and OUES); second, it compared HS and cycle ergometer ventilatory efficiency in constant-load tests conducted at an intensity equivalent to the LT. Although the cardioventilatory responses were greater in the cycle ergometer test as compared to HS, ventilatory efficiency was very similar between the two exercise modalities. In addition, the blood lactate concentrations were similar between both exercise modes although these values were slightly higher in HS exercise than in the cycle ergometer exercise at the end of the constant-load tests.

These findings replicated the results obtained in previous investigations, in which the cardiorespiratory responses were higher in the cycle ergometer test than in HS exercise [2]. The constant-load HS test at LT intensity likely induced a lower cardioventilatory response because a rest time was implemented between sets. To date, it has been unfeasible to perform a continuous protocol in the HS exercise at the LT intensity. In theory, a continuous HS protocol would increase intramuscular pressure leading to augmented muscle tension and progressive fatigue. These physiological mechanisms would produce the collapse of capillaries and diminish the oxygen available into the muscle and thus increasing the blood lactate levels [37]. Although it is usual to find a different cardiorespiratory response between several endurance exercise modalities at the same relative intensity [38], the available studies comparing resistance versus endurance exercises during constant-load test at LT intensity are currently insufficient to draw more precise conclusions.

The VE/VCO_2_ slope and OUES results obtained in both exercises are considered normal and comparable to other studies with healthy adults (19–30 in VE/VCO_2_ slope, 2.55 ± 1.01 n = 417 in OUES) [10, 16, 24]. In elite youth cyclists [11], the slope of the VE/VCO_2_ was similar (about 28) to our study, but the OUES was higher: 3.8 vs. 2.5 in our study. The difference could be due to the novel methodology used in our study and the greater cardiorespiratory fitness of elite youth cyclists. No studies are available for comparison of the VE/VCO_2_ slope, cycle ergometer values, or HS data in a constant-load test at LT intensity. However, our results on ventilatory efficiency were very similar to those obtained in other studies in endurance exercises (cycling) at the intensity of the anaerobic threshold [11], perhaps because both intensities (LT and anaerobic thresholds) reflect a similar metabolic moment, beyond which lactate concentrations begin to increase. Our data from both exercise modes in healthy young adults verify that this protocol could be another option to evaluate the slope of VE/VCO_2_ and OUES in a mostly aerobic metabolism, controlling the acidosis of the body, without having to reach the high intensities and avoiding a higher cardiorespiratory stress that could become problematic in some pathologies. Probably, during a constant-load test at moderate intensity (LT) the relationship between VE and both VCO_2_ and VO_2_ is normally stable and uniform before the onset of ventilatory compensation for the exercise-induced lactic acidosis [10], justifying, at least in part, the similarities detected in VE/VCO_2_ slope and OUES between both exercise modalities at the same metabolic state.

A surprising aspect of this study was the almost identical values in the ventilatory efficiency observed in the HS and cycle ergometer tests. Studies comparing the VE/VCO_2_ slope and the OUES in different types of exercises are rare; therefore, there is a significant lack of information about which exercise modality could induce a higher ventilatory efficiency. Our findings show that two types of exercise with different cardioventilatory responses induce the same ventilatory efficiency at similar metabolic intensity. During incremental exercise tests [23], no significant changes were found between treadmill and cycle ergometer trials, although both exercise modalities showed a lower VE/VCO_2_ slope (higher efficiency) compared to a robotics-assisted tilt table. A study compared OUES in 17 healthy subjects in two exercise modalities, observing higher values in the treadmill test compared to the cycle ergometer [24]. Although further evidence is needed, ventilatory efficiency could be dependent on the type of exercise, test protocol, and mode of assessing ventilatory efficiency (OUES vs VE/VCO_2_ slope).

It was expected that subjects with a lower VE/VCO_2_ slope (greater efficiency) throughout each of the tests would increase their OUES. The lack of significant correlation between the OUES and the slope of the VE/VCO_2_ in the two exercise modalities analyzed indicates that those subjects who showed greater ventilatory efficiency in the HS did not achieve greater ventilatory efficiency in the cycle ergometer. The OUES has been accepted as a valid submaximal measure of the function and prognosis of disease [39], and the slope of VE/VCO_2_ is a reliable assessment in healthy adults [40] and in those with pathologies [41]. However, their usefulness in healthy and athletically trained people is dubious. It is not yet clear which factors contribute to modify ventilatory efficiency during exercise, but the established postulates through this discussion may be more relevant in the clinical field than in fitness and sports performance because it seems that the VE/VCO_2_ slope did not change in elite cyclists after 16 weeks of training [11] and, regardless of gender, in children [42] and healthy adults [10] engaging in exercise. Training did not improve the OUES in healthy subjects [43] and could have a limited effect in athletes [11].

As a practical application, these findings could be an interesting alternative for the processes of physical rehabilitation and recovery from diseases associated with a loss of strength and muscle mass. For example, patients with heart failure are characterized by a significant loss of muscle mass, and these same physiological mechanisms are closely related to dyspnea and ventilatory fatigue [44]. Therefore, ventilatory efficiency is related to the severity of heart failure with reduced ejection fraction [45, 46] and, as a corollary, poor ventilatory efficiency is related to increased morbidity and mortality. In addition, it is common to diagnose strength and muscle loss (sarcopenia) in older adults. Sarcopenia is a prevalent syndrome associated with premature mortality in elderly [47]. Resistance exercises at LT intensity could increase local muscular endurance avoiding the losses of strength and muscular mass and, in addition, with the same ventilatory efficiency that could produce the cycle ergometer exercise. Unfortunately, our arguments cannot be consolidated with previous studies in different pathologies, as data clarifying the effect of the resistance exercises to LT intensity in patients with heart failure or sarcopenia are not available; therefore, these observations remain purely intuitive and speculative. It is clear that the combination of both resistance and endurance training has improved exercise capacity and diastolic function in patients with heart failure with reduced ejection fraction [48]. Accordingly, the combination of resistance exercises and endurance exercises could be an adequate methodology to increase cardiorespiratory response (endurance exercises) on the one hand and strength and muscular resistance (resistance exercises) on the other hand.

There are some limitations in this study with regard to the HS exercise protocol, which should be considered. The experimental procedures established in both incremental tests prompt controversial debate with regards to the location of the LT. Consequently, the relative intensity or external load prescribed in each exercise could have been different during both constant-load tests. In this case, an important bias would occur when comparing ventilatory efficiency, cardiorespiratory and metabolic responses between both exercises. However, the results reported by our research group in a recent study [49] revealed that the detection of the LT in both exercises using this same methodology could occur at a similar metabolic instant and relative intensity according to the criteria defined by Binder et al. [50]. In both incremental tests, an equivalent load intensity was produced at the LT, however, cardiorespiratory response was higher in cycle ergometer than in HS exercise during constant-load tests. It is habitual to observe an unequal cardioventilatory response when several exercise modes are compared at the same relative intensity or external load [51]. An identical trend was found in other studies that compared blood lactate, RER and cardiorespiratory responses in various exercises at lower and moderate intensities [38, 52]. Probably, cardiorespiratory responses are exercise mode-dependent at the same metabolic intensity and, therefore, these differences seem larger and more important to considerer at lighter and moderate intensities [38].

We cannot fail to mention that the recovery time established between each series is a key factor in maintaining low and stable levels of blood lactate in a primarily aerobic metabolism. It is assumed that this rest period would mainly affect the mechanisms of cardioventilatory recovery. However, our research group has observed in preliminary trials (unpublished data) that the combination of resistance exercises (in the form of circuit training), without rest between exercises, could keep blood lactate concentrations low and stable. The results stated in this study have important implications for our understanding of the load intensity and the recovery time that regulate ventilatory efficiency in a predominantly aerobic metabolism in HS exercise. Probably, a discontinuous constant-load HS test might induce a similar metabolic intensity and ventilatory efficiency as occurred during continuous constant-load cycle ergometer test. Further studies are needed to determine if the hypothetical increase in VO_2_ and ventilation associated with a continuous protocol without recovery time between series would increase ventilatory efficiency in the resistance exercises to LT intensity.

## Conclusions

Our findings showed that:

1. Cardioventilatory response was lower in HS exercise than in cycle ergometer during a constant-load test at LT intensity.
2. Ventilatory efficiency was equally efficient in the HS resistance exercise and in cycle ergometer exercise in a predominantly aerobic metabolism, which could have a significant impact in healthy people.
3. There was no correlation between the OUES and the slope of the VE/VCO_2_ in the two exercise modalities analyzed. Those subjects who showed greater ventilatory efficiency in the HS did not achieve higher ventilatory efficiency in the cycle ergometer.
4. Performing a constant-load HS protocol at LT intensity does not generate significant cardiorespiratory stress, while ventilatory efficiency is maintained and muscle strength and local muscular endurance, as well as gross mechanical efficiency, may improve according to previous findings of our research group.

Further research is needed to analyze ventilatory efficiency for better understanding of ventilatory mechanisms that conditioning resistance exercises performance in a predominantly aerobic metabolism.

## Supporting information

S1 File Statistical analysis performed with the data obtained during constant-load test. (DOC)

S1 Fig Results for the preparation of the figures. (XLSX)
