# Supplementary material for: Ventilatory efficiency during constant-load test at lactate threshold intensity: Endurance versus resistance exercises
Source: PLoS One. 2019 May 21;14(5):e0216824. doi: 10.1371/journal.pone.0216824 (PMC6528988; doi:10.1371/journal.pone.0216824)
Supplement: S1 File — (DOC) [file pone.0216824.s001.doc]

General linear model. Oxygen Uptake (VO2)


Intra-subject factors	
Medida:   MEASURE_1  	
Exercise_modality	Checkpoints	Dependient variable	
1	1	VO2_HS_P1	
	2	VO2_HS_P2	
	3	VO2_HS_P3	
	4	VO2_HS_P4	
	5	VO2_HS_P5	
	6	VO2_HS_P6	
	7	VO2_HS_P7	
2	1	VO2_CYC_P1	
	2	VO2_CYC_P2	
	3	VO2_CYC_P3	
	4	VO2_CYC_P4	
	5	VO2_CYC_P5	
	6	VO2_CYC_P6	
	7	VO2_CYC_P7	


Descriptive statistics	
	Media	Desv. Desviación	N	
VO2_HS_P1	1,5110	,19910	18	
VO2_HS_P2	1,5982	,19120	18	
VO2_HS_P3	1,6035	,23899	18	
VO2_HS_P4	1,5876	,19871	18	
VO2_HS_P5	1,6215	,20509	18	
VO2_HS_P6	1,6940	,17512	18	
VO2_HS_P7	1,6883	,22154	18	
VO2_CYC_P1	2,0402	,40944	18	
VO2_CYC_P2	2,2535	,50178	18	
VO2_CYC_P3	2,2656	,46901	18	
VO2_CYC_P4	2,2555	,44101	18	
VO2_CYC_P5	2,2861	,43952	18	
VO2_CYC_P6	2,2775	,42466	18	
VO2_CYC_P7	2,2852	,41860	18	


Intra-subject factors	
Medida:   MEASURE_1  	
Origen	Tipo III de suma de cuadrados	gl	Media cuadrática	F	Sig.	Eta parcial al cuadrado	Parámetro sin centralidad	Potencia observadaa	
Exercise_modality	Esfericidad asumida	24,436	1	24,436	29,971	,000	,638	29,971	,999	
	Greenhouse-Geisser	24,436	1,000	24,436	29,971	,000	,638	29,971	,999	
	Huynh-Feldt	24,436	1,000	24,436	29,971	,000	,638	29,971	,999	
	Límite inferior	24,436	1,000	24,436	29,971	,000	,638	29,971	,999	
Error(Exercise_modality)	Esfericidad asumida	13,861	17	,815						
	Greenhouse-Geisser	13,861	17,000	,815						
	Huynh-Feldt	13,861	17,000	,815						
	Límite inferior	13,861	17,000	,815						
Checkpoints	Esfericidad asumida	1,107	6	,185	21,390	,000	,557	128,340	1,000	
	Greenhouse-Geisser	1,107	3,013	,367	21,390	,000	,557	64,451	1,000	
	Huynh-Feldt	1,107	3,735	,296	21,390	,000	,557	79,884	1,000	
	Límite inferior	1,107	1,000	1,107	21,390	,000	,557	21,390	,992	
Error(Checkpoints)	Esfericidad asumida	,880	102	,009						
	Greenhouse-Geisser	,880	51,223	,017						
	Huynh-Feldt	,880	63,489	,014						
	Límite inferior	,880	17,000	,052						
Exercise_modality * Checkpoints	Esfericidad asumida	,156	6	,026	3,175	,007	,157	19,051	,909	
	Greenhouse-Geisser	,156	3,224	,048	3,175	,028	,157	10,238	,726	
	Huynh-Feldt	,156	4,068	,038	3,175	,018	,157	12,917	,802	
	Límite inferior	,156	1,000	,156	3,175	,093	,157	3,175	,390	
Error(Exercise_modality*Checkpoints)	Esfericidad asumida	,836	102	,008						
	Greenhouse-Geisser	,836	54,816	,015						
	Huynh-Feldt	,836	69,157	,012						
	Límite inferior	,836	17,000	,049						
a. Se ha calculado utilizando alpha =	


2. Exercise_modality

Estimates	
Medida:   MEASURE_1  	
Exercise_modality	Media	Desv. Error	Intervalo de confianza al 95%	
			Límite inferior	Límite superior	
1	1,615	,044	1,522	1,708	
2	2,238	,103	2,021	2,455	


Comparisons in pairs	
Medida:   MEASURE_1  	
(I) Exercise_modality	(J) Exercise_modality	Diferencia de medias (I-J)	Desv. Error	Sig.b	95% de intervalo de confianza para diferenciab	
					Límite inferior	Límite superior	
1	2	-,623*	,114	,000	-,863	-,383	
2	1	,623*	,114	,000	,383	,863	
Se basa en medias marginales estimadas	
*. La diferencia de medias es significativa en el nivel	
b. Ajuste para varias comparaciones: Bonferroni.	

3. Checkpoints

Estimates	
Medida:   MEASURE_1  	
Checkpoints	Media	Desv. Error	Intervalo de confianza al 95%	
			Límite inferior	Límite superior	
1	1,776	,047	1,676	1,875	
2	1,926	,065	1,789	2,063	
3	1,935	,065	1,798	2,071	
4	1,922	,055	1,805	2,038	
5	1,954	,057	1,834	2,074	
6	1,986	,050	1,880	2,092	
7	1,987	,056	1,869	2,105	


Comparisons in pairs	
Medida:   MEASURE_1  	
(I) Checkpoints	(J) Checkpoints	Diferencia de medias (I-J)	Desv. Error	Sig.b	95% de intervalo de confianza para diferenciab	
					Límite inferior	Límite superior	
1	2	-,150*	,029	,002	-,254	-,047	
	3	-,159*	,034	,004	-,279	-,039	
	4	-,146*	,025	,000	-,236	-,056	
	5	-,178*	,028	,000	-,277	-,080	
	6	-,210*	,021	,000	-,286	-,135	
	7	-,211*	,022	,000	-,289	-,134	
2	1	,150*	,029	,002	,047	,254	
	3	-,009	,014	1,000	-,059	,041	
	4	,004	,019	1,000	-,064	,072	
	5	-,028	,023	1,000	-,109	,053	
	6	-,060	,025	,558	-,148	,028	
	7	-,061	,019	,093	-,127	,005	
3	1	,159*	,034	,004	,039	,279	
	2	,009	,014	1,000	-,041	,059	
	4	,013	,019	1,000	-,055	,081	
	5	-,019	,023	1,000	-,101	,062	
	6	-,051	,028	1,000	-,150	,047	
	7	-,052	,022	,624	-,131	,026	
4	1	,146*	,025	,000	,056	,236	
	2	-,004	,019	1,000	-,072	,064	
	3	-,013	,019	1,000	-,081	,055	
	5	-,032	,011	,210	-,072	,007	
	6	-,064*	,015	,013	-,119	-,009	
	7	-,065	,019	,078	-,134	,004	
5	1	,178*	,028	,000	,080	,277	
	2	,028	,023	1,000	-,053	,109	
	3	,019	,023	1,000	-,062	,101	
	4	,032	,011	,210	-,007	,072	
	6	-,032	,017	1,000	-,092	,028	
	7	-,033	,016	1,000	-,092	,026	
6	1	,210*	,021	,000	,135	,286	
	2	,060	,025	,558	-,028	,148	
	3	,051	,028	1,000	-,047	,150	
	4	,064*	,015	,013	,009	,119	
	5	,032	,017	1,000	-,028	,092	
	7	-,001	,018	1,000	-,067	,065	
7	1	,211*	,022	,000	,134	,289	
	2	,061	,019	,093	-,005	,127	
	3	,052	,022	,624	-,026	,131	
	4	,065	,019	,078	-,004	,134	
	5	,033	,016	1,000	-,026	,092	
	6	,001	,018	1,000	-,065	,067	
Se basa en medias marginales estimadas	
*. La diferencia de medias es significativa en el nivel	
b. Ajuste para varias comparaciones: Bonferroni.	


4. Exercise_modality * Checkpoints	
Medida:   MEASURE_1  	
Exercise_modality	Checkpoints	Media	Desv. Error	Intervalo de confianza al 95%	
				Límite inferior	Límite superior	
1	1	1,511	,047	1,412	1,610	
	2	1,598	,045	1,503	1,693	
	3	1,603	,056	1,485	1,722	
	4	1,588	,047	1,489	1,686	
	5	1,621	,048	1,519	1,723	
	6	1,694	,041	1,607	1,781	
	7	1,688	,052	1,578	1,798	
2	1	2,040	,097	1,837	2,244	
	2	2,254	,118	2,004	2,503	
	3	2,266	,111	2,032	2,499	
	4	2,256	,104	2,036	2,475	
	5	2,286	,104	2,068	2,505	
	6	2,278	,100	2,066	2,489	
	7	2,285	,099	2,077	2,493	


5. Exercise_modality * Checkpoints

Estimates	
Medida:   MEASURE_1  	
Exercise_modality	Checkpoints	Media	Desv. Error	Intervalo de confianza al 95%	
				Límite inferior	Límite superior	
1	1	1,511	,047	1,412	1,610	
	2	1,598	,045	1,503	1,693	
	3	1,603	,056	1,485	1,722	
	4	1,588	,047	1,489	1,686	
	5	1,621	,048	1,519	1,723	
	6	1,694	,041	1,607	1,781	
	7	1,688	,052	1,578	1,798	
2	1	2,040	,097	1,837	2,244	
	2	2,254	,118	2,004	2,503	
	3	2,266	,111	2,032	2,499	
	4	2,256	,104	2,036	2,475	
	5	2,286	,104	2,068	2,505	
	6	2,278	,100	2,066	2,489	
	7	2,285	,099	2,077	2,493	


Comparisons in pairs	
Medida:   MEASURE_1  	
Checkpoints	(I) Exercise_modality	(J) Exercise_modality	Diferencia de medias (I-J)	Desv. Error	Sig.b	95% de intervalo de confianza para diferenciab	
						Límite inferior	Límite superior	
1	1	2	-,529*	,119	,000	-,779	-,279	
	2	1	,529*	,119	,000	,279	,779	
2	1	2	-,655*	,123	,000	-,915	-,396	
	2	1	,655*	,123	,000	,396	,915	
3	1	2	-,662*	,119	,000	-,913	-,411	
	2	1	,662*	,119	,000	,411	,913	
4	1	2	-,668*	,117	,000	-,915	-,420	
	2	1	,668*	,117	,000	,420	,915	
5	1	2	-,665*	,115	,000	-,907	-,423	
	2	1	,665*	,115	,000	,423	,907	
6	1	2	-,583*	,115	,000	-,827	-,340	
	2	1	,583*	,115	,000	,340	,827	
7	1	2	-,597*	,112	,000	-,833	-,361	
	2	1	,597*	,112	,000	,361	,833	
Se basa en medias marginales estimadas	
*. La diferencia de medias es significativa en el nivel	
b. Ajuste para varias comparaciones: Bonferroni.	


6. Exercise_modality * Checkpoints

Estimates	
Medida:   MEASURE_1  	
Exercise_modality	Checkpoints	Media	Desv. Error	Intervalo de confianza al 95%	
				Límite inferior	Límite superior	
1	1	1,511	,047	1,412	1,610	
	2	1,598	,045	1,503	1,693	
	3	1,603	,056	1,485	1,722	
	4	1,588	,047	1,489	1,686	
	5	1,621	,048	1,519	1,723	
	6	1,694	,041	1,607	1,781	
	7	1,688	,052	1,578	1,798	
2	1	2,040	,097	1,837	2,244	
	2	2,254	,118	2,004	2,503	
	3	2,266	,111	2,032	2,499	
	4	2,256	,104	2,036	2,475	
	5	2,286	,104	2,068	2,505	
	6	2,278	,100	2,066	2,489	
	7	2,285	,099	2,077	2,493	


Comparisons in pairs	
Medida:   MEASURE_1  	
Exercise_modality	(I) Checkpoints	(J) Checkpoints	Diferencia de medias (I-J)	Desv. Error	Sig.b	95% de intervalo de confianza para diferenciab	
						Límite inferior	Límite superior	
1	1	2	-,087*	,023	,035	-,171	-,004	
		3	-,092	,040	,719	-,236	,051	
		4	-,077	,029	,326	-,178	,025	
		5	-,111	,034	,100	-,232	,011	
		6	-,183*	,032	,001	-,299	-,067	
		7	-,177*	,037	,004	-,311	-,044	
	2	1	,087*	,023	,035	,004	,171	
		3	-,005	,028	1,000	-,106	,095	
		4	,011	,021	1,000	-,063	,084	
		5	-,023	,031	1,000	-,134	,088	
		6	-,096	,034	,244	-,217	,025	
		7	-,090	,030	,157	-,196	,016	
	3	1	,092	,040	,719	-,051	,236	
		2	,005	,028	1,000	-,095	,106	
		4	,016	,023	1,000	-,068	,099	
		5	-,018	,034	1,000	-,138	,102	
		6	-,091	,041	,893	-,238	,057	
		7	-,085	,029	,200	-,188	,019	
	4	1	,077	,029	,326	-,025	,178	
		2	-,011	,021	1,000	-,084	,063	
		3	-,016	,023	1,000	-,099	,068	
		5	-,034	,018	1,000	-,099	,031	
		6	-,106	,031	,074	-,218	,006	
		7	-,101*	,026	,026	-,194	-,008	
	5	1	,111	,034	,100	-,011	,232	
		2	,023	,031	1,000	-,088	,134	
		3	,018	,034	1,000	-,102	,138	
		4	,034	,018	1,000	-,031	,099	
		6	-,073	,032	,816	-,188	,043	
		7	-,067	,026	,419	-,160	,026	
	6	1	,183*	,032	,001	,067	,299	
		2	,096	,034	,244	-,025	,217	
		3	,091	,041	,893	-,057	,238	
		4	,106	,031	,074	-,006	,218	
		5	,073	,032	,816	-,043	,188	
		7	,006	,025	1,000	-,085	,096	
	7	1	,177*	,037	,004	,044	,311	
		2	,090	,030	,157	-,016	,196	
		3	,085	,029	,200	-,019	,188	
		4	,101*	,026	,026	,008	,194	
		5	,067	,026	,419	-,026	,160	
		6	-,006	,025	1,000	-,096	,085	
2	1	2	-,213*	,043	,002	-,367	-,060	
		3	-,225*	,039	,001	-,366	-,085	
		4	-,215*	,032	,000	-,328	-,103	
		5	-,246*	,031	,000	-,358	-,134	
		6	-,237*	,024	,000	-,321	-,153	
		7	-,245*	,027	,000	-,341	-,149	
	2	1	,213*	,043	,002	,060	,367	
		3	-,012	,015	1,000	-,066	,042	
		4	-,002	,033	1,000	-,120	,116	
		5	-,033	,035	1,000	-,158	,093	
		6	-,024	,035	1,000	-,150	,102	
		7	-,032	,041	1,000	-,179	,116	
	3	1	,225*	,039	,001	,085	,366	
		2	,012	,015	1,000	-,042	,066	
		4	,010	,023	1,000	-,072	,092	
		5	-,021	,027	1,000	-,116	,075	
		6	-,012	,029	1,000	-,117	,093	
		7	-,020	,038	1,000	-,155	,115	
	4	1	,215*	,032	,000	,103	,328	
		2	,002	,033	1,000	-,116	,120	
		3	-,010	,023	1,000	-,092	,072	
		5	-,031	,012	,510	-,075	,014	
		6	-,022	,022	1,000	-,099	,055	
		7	-,030	,038	1,000	-,164	,105	
	5	1	,246*	,031	,000	,134	,358	
		2	,033	,035	1,000	-,093	,158	
		3	,021	,027	1,000	-,075	,116	
		4	,031	,012	,510	-,014	,075	
		6	,009	,020	1,000	-,062	,079	
		7	,001	,035	1,000	-,125	,127	
	6	1	,237*	,024	,000	,153	,321	
		2	,024	,035	1,000	-,102	,150	
		3	,012	,029	1,000	-,093	,117	
		4	,022	,022	1,000	-,055	,099	
		5	-,009	,020	1,000	-,079	,062	
		7	-,008	,021	1,000	-,082	,067	
	7	1	,245*	,027	,000	,149	,341	
		2	,032	,041	1,000	-,116	,179	
		3	,020	,038	1,000	-,115	,155	
		4	,030	,038	1,000	-,105	,164	
		5	-,001	,035	1,000	-,127	,125	
		6	,008	,021	1,000	-,067	,082	
Se basa en medias marginales estimadas	
*. La diferencia de medias es significativa en el nivel	
b. Ajuste para varias comparaciones: Bonferroni.	


General linear model. Respiratory Exchange Ratio (RER)

Intra-subject factors	
Medida:   MEASURE_1  	
Exercise_modality	Checkpoints	Variable dependiente	
1	1	RER_HS_P1	
	2	RER_HS_P2	
	3	RER_HS_P3	
	4	RER_HS_P4	
	5	RER_HS_P5	
	6	RER_HS_P6	
	7	RER_HS_P7	
2	1	RER_CYC_P1	
	2	RER_CYC_P2	
	3	RER_CYC_P3	
	4	RER_CYC_P4	
	5	RER_CYC_P5	
	6	RER_CYC_P6	
	7	RER_CYC_P7	


Descriptive statistics	
	Media	Desv. Desviación	N	
RER_HS_P1	,9054	,05085	18	
RER_HS_P2	,9535	,04573	18	
RER_HS_P3	,9463	,04418	18	
RER_HS_P4	,9409	,04643	18	
RER_HS_P5	,9424	,05051	18	
RER_HS_P6	,9346	,04903	18	
RER_HS_P7	,9381	,04598	18	
RER_CYC_P1	,8881	,05464	18	
RER_CYC_P2	,9283	,04351	18	
RER_CYC_P3	,9272	,03451	18	
RER_CYC_P4	,9156	,03434	18	
RER_CYC_P5	,9137	,03533	18	
RER_CYC_P6	,9124	,03466	18	
RER_CYC_P7	,9120	,03541	18	


Intra-subject factors	
Medida:   MEASURE_1  	
Origen	Tipo III de suma de cuadrados	gl	Media cuadrática	F	Sig.	Eta parcial al cuadrado	Parámetro sin centralidad	Potencia observadaa	
Exercise_modality	Esfericidad asumida	,035	1	,035	3,358	,084	,165	3,358	,409	
	Greenhouse-Geisser	,035	1,000	,035	3,358	,084	,165	3,358	,409	
	Huynh-Feldt	,035	1,000	,035	3,358	,084	,165	3,358	,409	
	Límite inferior	,035	1,000	,035	3,358	,084	,165	3,358	,409	
Error(Exercise_modality)	Esfericidad asumida	,175	17	,010						
	Greenhouse-Geisser	,175	17,000	,010						
	Huynh-Feldt	,175	17,000	,010						
	Límite inferior	,175	17,000	,010						
Checkpoints	Esfericidad asumida	,044	6	,007	15,391	,000	,475	92,349	1,000	
	Greenhouse-Geisser	,044	2,782	,016	15,391	,000	,475	42,816	1,000	
	Huynh-Feldt	,044	3,381	,013	15,391	,000	,475	52,039	1,000	
	Límite inferior	,044	1,000	,044	15,391	,001	,475	15,391	,958	
Error(Checkpoints)	Esfericidad asumida	,048	102	,000						
	Greenhouse-Geisser	,048	47,291	,001						
	Huynh-Feldt	,048	57,478	,001						
	Límite inferior	,048	17,000	,003						
Exercise_modality * Checkpoints	Esfericidad asumida	,001	6	,000	,324	,923	,019	1,942	,137	
	Greenhouse-Geisser	,001	2,514	,000	,324	,773	,019	,814	,103	
	Huynh-Feldt	,001	2,986	,000	,324	,807	,019	,967	,108	
	Límite inferior	,001	1,000	,001	,324	,577	,019	,324	,084	
Error(Exercise_modality*Checkpoints)	Esfericidad asumida	,048	102	,000						
	Greenhouse-Geisser	,048	42,744	,001						
	Huynh-Feldt	,048	50,767	,001						
	Límite inferior	,048	17,000	,003						
a. Se ha calculado utilizando alpha =	


2. Exercise_modality


Estimates	
Medida:   MEASURE_1  	
Exercise_modality	Media	Desv. Error	Intervalo de confianza al 95%	
			Límite inferior	Límite superior	
1	,937	,010	,917	,958	
2	,914	,009	,896	,932	


Comparisons in pairs	
Medida:   MEASURE_1  	
(I) Exercise_modality	(J) Exercise_modality	Diferencia de medias (I-J)	Desv. Error	Sig.a	95% de intervalo de confianza para diferenciaa	
					Límite inferior	Límite superior	
1	2	,023	,013	,084	-,004	,050	
2	1	-,023	,013	,084	-,050	,004	
Se basa en medias marginales estimadas	
a. Ajuste para varias comparaciones: Bonferroni.	

3. Checkpoints

Estimates	
Medida:   MEASURE_1  	
Checkpoints	Media	Desv. Error	Intervalo de confianza al 95%	
			Límite inferior	Límite superior	
1	,897	,009	,878	,916	
2	,941	,007	,925	,956	
3	,937	,007	,922	,952	
4	,928	,007	,914	,942	
5	,928	,008	,912	,944	
6	,924	,007	,910	,937	
7	,925	,007	,910	,941	


Comparisons in pairs	
Medida:   MEASURE_1  	
(I) Checkpoints	(J) Checkpoints	Diferencia de medias (I-J)	Desv. Error	Sig.b	95% de intervalo de confianza para diferenciab	
					Límite inferior	Límite superior	
1	2	-,044*	,005	,000	-,063	-,025	
	3	-,040*	,006	,000	-,063	-,017	
	4	-,031*	,006	,003	-,055	-,008	
	5	-,031*	,007	,004	-,055	-,008	
	6	-,027*	,007	,034	-,052	-,001	
	7	-,028	,008	,077	-,058	,002	
2	1	,044*	,005	,000	,025	,063	
	3	,004	,004	1,000	-,009	,017	
	4	,013	,005	,331	-,004	,030	
	5	,013	,005	,416	-,005	,031	
	6	,017	,005	,078	-,001	,036	
	7	,016	,006	,287	-,005	,036	
3	1	,040*	,006	,000	,017	,063	
	2	-,004	,004	1,000	-,017	,009	
	4	,009	,003	,178	-,002	,019	
	5	,009	,005	1,000	-,007	,025	
	6	,013	,005	,208	-,003	,030	
	7	,012	,005	,452	-,005	,028	
4	1	,031*	,006	,003	,008	,055	
	2	-,013	,005	,331	-,030	,004	
	3	-,009	,003	,178	-,019	,002	
	5	,000	,004	1,000	-,013	,013	
	6	,005	,004	1,000	-,009	,018	
	7	,003	,004	1,000	-,010	,017	
5	1	,031*	,007	,004	,008	,055	
	2	-,013	,005	,416	-,031	,005	
	3	-,009	,005	1,000	-,025	,007	
	4	,000	,004	1,000	-,013	,013	
	6	,005	,004	1,000	-,008	,017	
	7	,003	,004	1,000	-,013	,019	
6	1	,027*	,007	,034	,001	,052	
	2	-,017	,005	,078	-,036	,001	
	3	-,013	,005	,208	-,030	,003	
	4	-,005	,004	1,000	-,018	,009	
	5	-,005	,004	1,000	-,017	,008	
	7	-,002	,003	1,000	-,012	,009	
7	1	,028	,008	,077	-,002	,058	
	2	-,016	,006	,287	-,036	,005	
	3	-,012	,005	,452	-,028	,005	
	4	-,003	,004	1,000	-,017	,010	
	5	-,003	,004	1,000	-,019	,013	
	6	,002	,003	1,000	-,009	,012	
Se basa en medias marginales estimadas	
*. La diferencia de medias es significativa en el nivel	
b. Ajuste para varias comparaciones: Bonferroni.	


4. Exercise_modality * Checkpoints	
Medida:   MEASURE_1  	
Exercise_modality	Checkpoints	Media	Desv. Error	Intervalo de confianza al 95%	
				Límite inferior	Límite superior	
1	1	,905	,012	,880	,931	
	2	,954	,011	,931	,976	
	3	,946	,010	,924	,968	
	4	,941	,011	,918	,964	
	5	,942	,012	,917	,968	
	6	,935	,012	,910	,959	
	7	,938	,011	,915	,961	
2	1	,888	,013	,861	,915	
	2	,928	,010	,907	,950	
	3	,927	,008	,910	,944	
	4	,916	,008	,898	,933	
	5	,914	,008	,896	,931	
	6	,912	,008	,895	,930	
	7	,912	,008	,894	,930	


5. Exercise_modality * Checkpoints


Estimates	
Medida:   MEASURE_1  	
Exercise_modality	Checkpoints	Media	Desv. Error	Intervalo de confianza al 95%	
				Límite inferior	Límite superior	
1	1	,905	,012	,880	,931	
	2	,954	,011	,931	,976	
	3	,946	,010	,924	,968	
	4	,941	,011	,918	,964	
	5	,942	,012	,917	,968	
	6	,935	,012	,910	,959	
	7	,938	,011	,915	,961	
2	1	,888	,013	,861	,915	
	2	,928	,010	,907	,950	
	3	,927	,008	,910	,944	
	4	,916	,008	,898	,933	
	5	,914	,008	,896	,931	
	6	,912	,008	,895	,930	
	7	,912	,008	,894	,930	


Comparisons in pairs	
Medida:   MEASURE_1  	
Checkpoints	(I) Exercise_modality	(J) Exercise_modality	Diferencia de medias (I-J)	Desv. Error	Sig.a	95% de intervalo de confianza para diferenciaa	
						Límite inferior	Límite superior	
1	1	2	,017	,017	,334	-,019	,054	
	2	1	-,017	,017	,334	-,054	,019	
2	1	2	,025	,015	,114	-,007	,057	
	2	1	-,025	,015	,114	-,057	,007	
3	1	2	,019	,012	,141	-,007	,045	
	2	1	-,019	,012	,141	-,045	,007	
4	1	2	,025	,014	,084	-,004	,055	
	2	1	-,025	,014	,084	-,055	,004	
5	1	2	,029	,014	,055	-,001	,058	
	2	1	-,029	,014	,055	-,058	,001	
6	1	2	,022	,015	,160	-,010	,054	
	2	1	-,022	,015	,160	-,054	,010	
7	1	2	,026	,013	,055	-,001	,053	
	2	1	-,026	,013	,055	-,053	,001	
Se basa en medias marginales estimadas	
a. Ajuste para varias comparaciones: Bonferroni.	


6. Exercise_modality * Checkpoints


Estimates	
Medida:   MEASURE_1  	
Exercise_modality	Checkpoints	Media	Desv. Error	Intervalo de confianza al 95%	
				Límite inferior	Límite superior	
1	1	,905	,012	,880	,931	
	2	,954	,011	,931	,976	
	3	,946	,010	,924	,968	
	4	,941	,011	,918	,964	
	5	,942	,012	,917	,968	
	6	,935	,012	,910	,959	
	7	,938	,011	,915	,961	
2	1	,888	,013	,861	,915	
	2	,928	,010	,907	,950	
	3	,927	,008	,910	,944	
	4	,916	,008	,898	,933	
	5	,914	,008	,896	,931	
	6	,912	,008	,895	,930	
	7	,912	,008	,894	,930	


Comparisons in pairs	
Medida:   MEASURE_1  	
Exercise_modality	(I) Checkpoints	(J) Checkpoints	Diferencia de medias (I-J)	Desv. Error	Sig.b	95% de intervalo de confianza para diferenciab	
						Límite inferior	Límite superior	
1	1	2	-,048*	,008	,000	-,076	-,021	
		3	-,041*	,011	,031	-,079	-,002	
		4	-,036*	,010	,043	-,070	-,001	
		5	-,037	,012	,163	-,081	,007	
		6	-,029	,012	,466	-,071	,012	
		7	-,033	,012	,368	-,077	,012	
	2	1	,048*	,008	,000	,021	,076	
		3	,007	,007	1,000	-,016	,031	
		4	,013	,007	1,000	-,011	,036	
		5	,011	,009	1,000	-,021	,043	
		6	,019	,008	,762	-,011	,049	
		7	,015	,008	1,000	-,014	,045	
	3	1	,041*	,011	,031	,002	,079	
		2	-,007	,007	1,000	-,031	,016	
		4	,005	,005	1,000	-,012	,022	
		5	,004	,008	1,000	-,023	,031	
		6	,012	,009	1,000	-,021	,044	
		7	,008	,008	1,000	-,021	,037	
	4	1	,036*	,010	,043	,001	,070	
		2	-,013	,007	1,000	-,036	,011	
		3	-,005	,005	1,000	-,022	,012	
		5	-,001	,007	1,000	-,025	,022	
		6	,006	,007	1,000	-,019	,031	
		7	,003	,007	1,000	-,020	,026	
	5	1	,037	,012	,163	-,007	,081	
		2	-,011	,009	1,000	-,043	,021	
		3	-,004	,008	1,000	-,031	,023	
		4	,001	,007	1,000	-,022	,025	
		6	,008	,006	1,000	-,014	,029	
		7	,004	,006	1,000	-,016	,024	
	6	1	,029	,012	,466	-,012	,071	
		2	-,019	,008	,762	-,049	,011	
		3	-,012	,009	1,000	-,044	,021	
		4	-,006	,007	1,000	-,031	,019	
		5	-,008	,006	1,000	-,029	,014	
		7	-,004	,005	1,000	-,020	,013	
	7	1	,033	,012	,368	-,012	,077	
		2	-,015	,008	1,000	-,045	,014	
		3	-,008	,008	1,000	-,037	,021	
		4	-,003	,007	1,000	-,026	,020	
		5	-,004	,006	1,000	-,024	,016	
		6	,004	,005	1,000	-,013	,020	
2	1	2	-,040*	,006	,000	-,060	-,020	
		3	-,039*	,007	,001	-,064	-,014	
		4	-,027*	,007	,030	-,053	-,002	
		5	-,026*	,007	,024	-,049	-,002	
		6	-,024	,008	,182	-,053	,005	
		7	-,024	,010	,663	-,060	,012	
	2	1	,040*	,006	,000	,020	,060	
		3	,001	,004	1,000	-,012	,015	
		4	,013	,005	,310	-,004	,030	
		5	,015	,005	,115	-,002	,031	
		6	,016	,007	,710	-,009	,041	
		7	,016	,008	1,000	-,014	,046	
	3	1	,039*	,007	,001	,014	,064	
		2	-,001	,004	1,000	-,015	,012	
		4	,012	,003	,072	-,001	,024	
		5	,014*	,004	,035	,001	,026	
		6	,015	,005	,168	-,003	,032	
		7	,015	,006	,565	-,007	,038	
	4	1	,027*	,007	,030	,002	,053	
		2	-,013	,005	,310	-,030	,004	
		3	-,012	,003	,072	-,024	,001	
		5	,002	,003	1,000	-,008	,011	
		6	,003	,004	1,000	-,010	,016	
		7	,004	,005	1,000	-,015	,022	
	5	1	,026*	,007	,024	,002	,049	
		2	-,015	,005	,115	-,031	,002	
		3	-,014*	,004	,035	-,026	-,001	
		4	-,002	,003	1,000	-,011	,008	
		6	,001	,004	1,000	-,014	,017	
		7	,002	,006	1,000	-,019	,022	
	6	1	,024	,008	,182	-,005	,053	
		2	-,016	,007	,710	-,041	,009	
		3	-,015	,005	,168	-,032	,003	
		4	-,003	,004	1,000	-,016	,010	
		5	-,001	,004	1,000	-,017	,014	
		7	,000	,003	1,000	-,011	,012	
	7	1	,024	,010	,663	-,012	,060	
		2	-,016	,008	1,000	-,046	,014	
		3	-,015	,006	,565	-,038	,007	
		4	-,004	,005	1,000	-,022	,015	
		5	-,002	,006	1,000	-,022	,019	
		6	,000	,003	1,000	-,012	,011	
Se basa en medias marginales estimadas	
*. La diferencia de medias es significativa en el nivel	
b. Ajuste para varias comparaciones: Bonferroni.	


General linear model. Heart rate (HR)

Intra-subject factors	
Medida:   MEASURE_1  	
Exercise_modality	Checkpoints	Variable dependiente	
1	1	HS_HR_P1	
	2	HS_HR_P2	
	3	HS_HR_P3	
	4	HS_HR_P4	
	5	HS_HR_P5	
	6	HS_HR_P6	
	7	HS_HR_P7	
2	1	CYC_HR_P1	
	2	CYC_HR_P2	
	3	CYC_HR_P3	
	4	CYC_HR_P4	
	5	CYC_HR_P5	
	6	CYC_HR_P6	
	7	CYC_HR_P7	


Descriptive statistics	
	Media	Desv. Desviación	N	
HS_HR_P1	120,2963	14,61975	18	
HS_HR_P2	121,3519	15,94372	18	
HS_HR_P3	122,4259	14,62195	18	
HS_HR_P4	123,2222	13,89550	18	
HS_HR_P5	124,8148	14,70104	18	
HS_HR_P6	126,9630	13,88369	18	
HS_HR_P7	127,1667	16,46079	18	
CYC_HR_P1	127,5833	15,22192	18	
CYC_HR_P2	137,4352	16,32254	18	
CYC_HR_P3	140,5926	19,58576	18	
CYC_HR_P4	141,0833	17,35380	18	
CYC_HR_P5	142,5185	17,19894	18	
CYC_HR_P6	143,9074	16,79220	18	
CYC_HR_P7	144,1111	19,55711	18	


Intra-subject factors	
Medida:   MEASURE_1  	
Origen	Tipo III de suma de cuadrados	gl	Media cuadrática	F	Sig.	Eta parcial al cuadrado	Parámetro sin centralidad	Potencia observadaa	
Exercise_modality	Esfericidad asumida	15838,643	1	15838,643	21,915	,000	,563	21,915	,993	
	Greenhouse-Geisser	15838,643	1,000	15838,643	21,915	,000	,563	21,915	,993	
	Huynh-Feldt	15838,643	1,000	15838,643	21,915	,000	,563	21,915	,993	
	Límite inferior	15838,643	1,000	15838,643	21,915	,000	,563	21,915	,993	
Error(Exercise_modality)	Esfericidad asumida	12286,272	17	722,722						
	Greenhouse-Geisser	12286,272	17,000	722,722						
	Huynh-Feldt	12286,272	17,000	722,722						
	Límite inferior	12286,272	17,000	722,722						
Checkpoints	Esfericidad asumida	3568,036	6	594,673	26,653	,000	,611	159,915	1,000	
	Greenhouse-Geisser	3568,036	2,347	1520,421	26,653	,000	,611	62,547	1,000	
	Huynh-Feldt	3568,036	2,746	1299,244	26,653	,000	,611	73,194	1,000	
	Límite inferior	3568,036	1,000	3568,036	26,653	,000	,611	26,653	,998	
Error(Checkpoints)	Esfericidad asumida	2275,829	102	22,312						
	Greenhouse-Geisser	2275,829	39,895	57,046						
	Huynh-Feldt	2275,829	46,686	48,747						
	Límite inferior	2275,829	17,000	133,872						
Exercise_modality * Checkpoints	Esfericidad asumida	797,597	6	132,933	5,846	,000	,256	35,073	,997	
	Greenhouse-Geisser	797,597	2,437	327,303	5,846	,004	,256	14,245	,894	
	Huynh-Feldt	797,597	2,875	277,460	5,846	,002	,256	16,804	,930	
	Límite inferior	797,597	1,000	797,597	5,846	,027	,256	5,846	,625	
Error(Exercise_modality*Checkpoints)	Esfericidad asumida	2319,586	102	22,741						
	Greenhouse-Geisser	2319,586	41,427	55,992						
	Huynh-Feldt	2319,586	48,869	47,466						
	Límite inferior	2319,586	17,000	136,446						
a. Se ha calculado utilizando alpha =	


2. Exercise_modality

Estimates	
Medida:   MEASURE_1  	
Exercise_modality	Media	Desv. Error	Intervalo de confianza al 95%	
			Límite inferior	Límite superior	
1	123,749	3,357	116,667	130,831	
2	139,604	3,992	131,182	148,027	


Comparisons in pairs	
Medida:   MEASURE_1  	
(I) Exercise_modality	(J) Exercise_modality	Diferencia de medias (I-J)	Desv. Error	Sig.b	95% de intervalo de confianza para diferenciab	
					Límite inferior	Límite superior	
1	2	-15,856*	3,387	,000	-23,002	-8,710	
2	1	15,856*	3,387	,000	8,710	23,002	
Se basa en medias marginales estimadas	
*. La diferencia de medias es significativa en el nivel	
b. Ajuste para varias comparaciones: Bonferroni.	


3. Checkpoints

Estimates	
Medida:   MEASURE_1  	
Checkpoints	Media	Desv. Error	Intervalo de confianza al 95%	
			Límite inferior	Límite superior	
1	123,940	3,159	117,274	130,605	
2	129,394	3,407	122,206	136,581	
3	131,509	3,525	124,071	138,947	
4	132,153	3,287	125,218	139,087	
5	133,667	3,281	126,744	140,590	
6	135,435	3,200	128,684	142,187	
7	135,639	3,609	128,024	143,253	


Comparisons in pairs	
Medida:   MEASURE_1  	
(I) Checkpoints	(J) Checkpoints	Diferencia de medias (I-J)	Desv. Error	Sig.b	95% de intervalo de confianza para diferenciab	
					Límite inferior	Límite superior	
1	2	-5,454*	,697	,000	-7,939	-2,968	
	3	-7,569*	1,160	,000	-11,707	-3,432	
	4	-8,213*	1,223	,000	-12,573	-3,853	
	5	-9,727*	1,294	,000	-14,339	-5,114	
	6	-11,495*	1,485	,000	-16,791	-6,200	
	7	-11,699*	1,748	,000	-17,933	-5,465	
2	1	5,454*	,697	,000	2,968	7,939	
	3	-2,116	,982	,964	-5,619	1,387	
	4	-2,759	1,123	,526	-6,763	1,245	
	5	-4,273*	1,193	,048	-8,527	-,019	
	6	-6,042*	1,403	,010	-11,044	-1,040	
	7	-6,245*	1,628	,028	-12,050	-,441	
3	1	7,569*	1,160	,000	3,432	11,707	
	2	2,116	,982	,964	-1,387	5,619	
	4	-,644	,744	1,000	-3,296	2,009	
	5	-2,157	,814	,354	-5,061	,746	
	6	-3,926*	1,066	,039	-7,727	-,125	
	7	-4,130*	,966	,011	-7,574	-,685	
4	1	8,213*	1,223	,000	3,853	12,573	
	2	2,759	1,123	,526	-1,245	6,763	
	3	,644	,744	1,000	-2,009	3,296	
	5	-1,514	,672	,791	-3,909	,881	
	6	-3,282	,966	,072	-6,727	,162	
	7	-3,486*	,913	,029	-6,740	-,232	
5	1	9,727*	1,294	,000	5,114	14,339	
	2	4,273*	1,193	,048	,019	8,527	
	3	2,157	,814	,354	-,746	5,061	
	4	1,514	,672	,791	-,881	3,909	
	6	-1,769	,655	,320	-4,105	,568	
	7	-1,972	,809	,546	-4,856	,911	
6	1	11,495*	1,485	,000	6,200	16,791	
	2	6,042*	1,403	,010	1,040	11,044	
	3	3,926*	1,066	,039	,125	7,727	
	4	3,282	,966	,072	-,162	6,727	
	5	1,769	,655	,320	-,568	4,105	
	7	-,204	,954	1,000	-3,606	3,199	
7	1	11,699*	1,748	,000	5,465	17,933	
	2	6,245*	1,628	,028	,441	12,050	
	3	4,130*	,966	,011	,685	7,574	
	4	3,486*	,913	,029	,232	6,740	
	5	1,972	,809	,546	-,911	4,856	
	6	,204	,954	1,000	-3,199	3,606	
Se basa en medias marginales estimadas	
*. La diferencia de medias es significativa en el nivel	
b. Ajuste para varias comparaciones: Bonferroni.	


4. Exercise_modality * Checkpoints	
Medida:   MEASURE_1  	
Exercise_modality	Checkpoints	Media	Desv. Error	Intervalo de confianza al 95%	
				Límite inferior	Límite superior	
1	1	120,296	3,446	113,026	127,567	
	2	121,352	3,758	113,423	129,280	
	3	122,426	3,446	115,155	129,697	
	4	123,222	3,275	116,312	130,132	
	5	124,815	3,465	117,504	132,125	
	6	126,963	3,272	120,059	133,867	
	7	127,167	3,880	118,981	135,352	
2	1	127,583	3,588	120,014	135,153	
	2	137,435	3,847	129,318	145,552	
	3	140,593	4,616	130,853	150,332	
	4	141,083	4,090	132,453	149,713	
	5	142,519	4,054	133,966	151,071	
	6	143,907	3,958	135,557	152,258	
	7	144,111	4,610	134,386	153,837	


5. Exercise_modality * Checkpoints


Estimates	
Medida:   MEASURE_1  	
Exercise_modality	Checkpoints	Media	Desv. Error	Intervalo de confianza al 95%	
				Límite inferior	Límite superior	
1	1	120,296	3,446	113,026	127,567	
	2	121,352	3,758	113,423	129,280	
	3	122,426	3,446	115,155	129,697	
	4	123,222	3,275	116,312	130,132	
	5	124,815	3,465	117,504	132,125	
	6	126,963	3,272	120,059	133,867	
	7	127,167	3,880	118,981	135,352	
2	1	127,583	3,588	120,014	135,153	
	2	137,435	3,847	129,318	145,552	
	3	140,593	4,616	130,853	150,332	
	4	141,083	4,090	132,453	149,713	
	5	142,519	4,054	133,966	151,071	
	6	143,907	3,958	135,557	152,258	
	7	144,111	4,610	134,386	153,837	


Comparisons in pairs	
Medida:   MEASURE_1  	
Checkpoints	(I) Exercise_modality	(J) Exercise_modality	Diferencia de medias (I-J)	Desv. Error	Sig.b	95% de intervalo de confianza para diferenciab	
						Límite inferior	Límite superior	
1	1	2	-7,287*	3,093	,031	-13,813	-,761	
	2	1	7,287*	3,093	,031	,761	13,813	
2	1	2	-16,083*	3,379	,000	-23,213	-8,954	
	2	1	16,083*	3,379	,000	8,954	23,213	
3	1	2	-18,167*	4,082	,000	-26,780	-9,554	
	2	1	18,167*	4,082	,000	9,554	26,780	
4	1	2	-17,861*	3,421	,000	-25,079	-10,643	
	2	1	17,861*	3,421	,000	10,643	25,079	
5	1	2	-17,704*	3,717	,000	-25,545	-9,862	
	2	1	17,704*	3,717	,000	9,862	25,545	
6	1	2	-16,944*	3,433	,000	-24,188	-9,701	
	2	1	16,944*	3,433	,000	9,701	24,188	
7	1	2	-16,944*	4,528	,002	-26,498	-7,391	
	2	1	16,944*	4,528	,002	7,391	26,498	
Se basa en medias marginales estimadas	
*. La diferencia de medias es significativa en el nivel	
b. Ajuste para varias comparaciones: Bonferroni.	

6. Exercise_modality * Checkpoints

Estimates	
Medida:   MEASURE_1  	
Exercise_modality	Checkpoints	Media	Desv. Error	Intervalo de confianza al 95%	
				Límite inferior	Límite superior	
1	1	120,296	3,446	113,026	127,567	
	2	121,352	3,758	113,423	129,280	
	3	122,426	3,446	115,155	129,697	
	4	123,222	3,275	116,312	130,132	
	5	124,815	3,465	117,504	132,125	
	6	126,963	3,272	120,059	133,867	
	7	127,167	3,880	118,981	135,352	
2	1	127,583	3,588	120,014	135,153	
	2	137,435	3,847	129,318	145,552	
	3	140,593	4,616	130,853	150,332	
	4	141,083	4,090	132,453	149,713	
	5	142,519	4,054	133,966	151,071	
	6	143,907	3,958	135,557	152,258	
	7	144,111	4,610	134,386	153,837	


Comparisons in pairs	
Medida:   MEASURE_1  	
Exercise_modality	(I) Checkpoints	(J) Checkpoints	Diferencia de medias (I-J)	Desv. Error	Sig.b	95% de intervalo de confianza para diferenciab	
						Límite inferior	Límite superior	
1	1	2	-1,056	1,057	1,000	-4,825	2,714	
		3	-2,130	1,502	1,000	-7,485	3,226	
		4	-2,926	1,640	1,000	-8,775	2,923	
		5	-4,519	2,063	,898	-11,874	2,837	
		6	-6,667	2,401	,272	-15,228	1,895	
		7	-6,870	2,144	,109	-14,513	,772	
	2	1	1,056	1,057	1,000	-2,714	4,825	
		3	-1,074	,929	1,000	-4,387	2,238	
		4	-1,870	1,386	1,000	-6,812	3,071	
		5	-3,463	1,693	1,000	-9,498	2,572	
		6	-5,611	2,164	,398	-13,327	2,104	
		7	-5,815	1,770	,092	-12,125	,495	
	3	1	2,130	1,502	1,000	-3,226	7,485	
		2	1,074	,929	1,000	-2,238	4,387	
		4	-,796	1,135	1,000	-4,842	3,250	
		5	-2,389	1,476	1,000	-7,653	2,875	
		6	-4,537	1,793	,453	-10,932	1,857	
		7	-4,741	1,486	,112	-10,038	,557	
	4	1	2,926	1,640	1,000	-2,923	8,775	
		2	1,870	1,386	1,000	-3,071	6,812	
		3	,796	1,135	1,000	-3,250	4,842	
		5	-1,593	1,132	1,000	-5,629	2,444	
		6	-3,741	1,535	,549	-9,215	1,734	
		7	-3,944	1,274	,138	-8,488	,599	
	5	1	4,519	2,063	,898	-2,837	11,874	
		2	3,463	1,693	1,000	-2,572	9,498	
		3	2,389	1,476	1,000	-2,875	7,653	
		4	1,593	1,132	1,000	-2,444	5,629	
		6	-2,148	1,157	1,000	-6,274	1,978	
		7	-2,352	1,133	1,000	-6,391	1,688	
	6	1	6,667	2,401	,272	-1,895	15,228	
		2	5,611	2,164	,398	-2,104	13,327	
		3	4,537	1,793	,453	-1,857	10,932	
		4	3,741	1,535	,549	-1,734	9,215	
		5	2,148	1,157	1,000	-1,978	6,274	
		7	-,204	1,256	1,000	-4,683	4,275	
	7	1	6,870	2,144	,109	-,772	14,513	
		2	5,815	1,770	,092	-,495	12,125	
		3	4,741	1,486	,112	-,557	10,038	
		4	3,944	1,274	,138	-,599	8,488	
		5	2,352	1,133	1,000	-1,688	6,391	
		6	,204	1,256	1,000	-4,275	4,683	
2	1	2	-9,852*	,901	,000	-13,063	-6,640	
		3	-13,009*	1,987	,000	-20,094	-5,925	
		4	-13,500*	1,596	,000	-19,190	-7,810	
		5	-14,935*	1,707	,000	-21,022	-8,848	
		6	-16,324*	1,647	,000	-22,197	-10,451	
		7	-16,528*	2,803	,000	-26,522	-6,534	
	2	1	9,852*	,901	,000	6,640	13,063	
		3	-3,157	1,656	1,000	-9,062	2,747	
		4	-3,648	1,398	,384	-8,632	1,336	
		5	-5,083	1,629	,131	-10,893	,726	
		6	-6,472*	1,589	,017	-12,137	-,808	
		7	-6,676	2,617	,434	-16,008	2,656	
	3	1	13,009*	1,987	,000	5,925	20,094	
		2	3,157	1,656	1,000	-2,747	9,062	
		4	-,491	1,227	1,000	-4,866	3,885	
		5	-1,926	1,365	1,000	-6,793	2,942	
		6	-3,315	1,454	,752	-8,499	1,869	
		7	-3,519	1,831	1,000	-10,048	3,011	
	4	1	13,500*	1,596	,000	7,810	19,190	
		2	3,648	1,398	,384	-1,336	8,632	
		3	,491	1,227	1,000	-3,885	4,866	
		5	-1,435	,477	,166	-3,137	,266	
		6	-2,824*	,693	,017	-5,295	-,354	
		7	-3,028	1,411	,978	-8,058	2,002	
	5	1	14,935*	1,707	,000	8,848	21,022	
		2	5,083	1,629	,131	-,726	10,893	
		3	1,926	1,365	1,000	-2,942	6,793	
		4	1,435	,477	,166	-,266	3,137	
		6	-1,389	,411	,075	-2,854	,076	
		7	-1,593	1,248	1,000	-6,043	2,858	
	6	1	16,324*	1,647	,000	10,451	22,197	
		2	6,472*	1,589	,017	,808	12,137	
		3	3,315	1,454	,752	-1,869	8,499	
		4	2,824*	,693	,017	,354	5,295	
		5	1,389	,411	,075	-,076	2,854	
		7	-,204	1,357	1,000	-5,043	4,636	
	7	1	16,528*	2,803	,000	6,534	26,522	
		2	6,676	2,617	,434	-2,656	16,008	
		3	3,519	1,831	1,000	-3,011	10,048	
		4	3,028	1,411	,978	-2,002	8,058	
		5	1,593	1,248	1,000	-2,858	6,043	
		6	,204	1,357	1,000	-4,636	5,043	
Se basa en medias marginales estimadas	
*. La diferencia de medias es significativa en el nivel	
b. Ajuste para varias comparaciones: Bonferroni.	


General linear model. ﻿Carbon dioxide (VCO2)


Intra-subject factors	
Medida:   MEASURE_1  	
Exercise_modality	Checkpoints	Variable dependiente	
1	1	HS_VCO2_P1	
	2	HS_VCO2_P2	
	3	HS_VCO2_P3	
	4	HS_VCO2_P4	
	5	HS_VCO2_P5	
	6	HS_VCO2_P6	
	7	HS_VCO2_P7	
2	1	CYC_VCO2_P1	
	2	CYC_VCO2_P2	
	3	CYC_VCO2_P3	
	4	CYC_VCO2_P4	
	5	CYC_VCO2_P5	
	6	CYC_VCO2_P6	
	7	CYC_VCO2_P7	


Descriptive statistics	
	Media	Desv. Desviación	N	
HS_VCO2_P1	1,3545	,21395	18	
HS_VCO2_P2	1,4968	,22171	18	
HS_VCO2_P3	1,5017	,25440	18	
HS_VCO2_P4	1,5001	,19741	18	
HS_VCO2_P5	1,5203	,21104	18	
HS_VCO2_P6	1,5571	,20457	18	
HS_VCO2_P7	1,5436	,18952	18	
CYC_VCO2_P1	1,8163	,39586	18	
CYC_VCO2_P2	2,0971	,48832	18	
CYC_VCO2_P3	2,0956	,45681	18	
CYC_VCO2_P4	2,0576	,42364	18	
CYC_VCO2_P5	2,0821	,42379	18	
CYC_VCO2_P6	2,0959	,41097	18	
CYC_VCO2_P7	2,0806	,40167	18	


Intra-subject factors	
Medida:   MEASURE_1  	
Origen	Tipo III de suma de cuadrados	gl	Media cuadrática	F	Sig.	Eta parcial al cuadrado	Parámetro sin centralidad	Potencia observadaa	
Exercise_modality	Esfericidad asumida	19,070	1	19,070	27,838	,000	,621	27,838	,999	
	Greenhouse-Geisser	19,070	1,000	19,070	27,838	,000	,621	27,838	,999	
	Huynh-Feldt	19,070	1,000	19,070	27,838	,000	,621	27,838	,999	
	Límite inferior	19,070	1,000	19,070	27,838	,000	,621	27,838	,999	
Error(Exercise_modality)	Esfericidad asumida	11,646	17	,685						
	Greenhouse-Geisser	11,646	17,000	,685						
	Huynh-Feldt	11,646	17,000	,685						
	Límite inferior	11,646	17,000	,685						
Checkpoints	Esfericidad asumida	1,499	6	,250	25,987	,000	,605	155,923	1,000	
	Greenhouse-Geisser	1,499	2,857	,525	25,987	,000	,605	74,242	1,000	
	Huynh-Feldt	1,499	3,495	,429	25,987	,000	,605	90,813	1,000	
	Límite inferior	1,499	1,000	1,499	25,987	,000	,605	25,987	,998	
Error(Checkpoints)	Esfericidad asumida	,980	102	,010						
	Greenhouse-Geisser	,980	48,567	,020						
	Huynh-Feldt	,980	59,407	,017						
	Límite inferior	,980	17,000	,058						
Exercise_modality * Checkpoints	Esfericidad asumida	,114	6	,019	2,082	,062	,109	12,495	,728	
	Greenhouse-Geisser	,114	2,941	,039	2,082	,115	,109	6,124	,496	
	Huynh-Feldt	,114	3,623	,032	2,082	,100	,109	7,545	,559	
	Límite inferior	,114	1,000	,114	2,082	,167	,109	2,082	,275	
Error(Exercise_modality*Checkpoints)	Esfericidad asumida	,935	102	,009						
	Greenhouse-Geisser	,935	49,994	,019						
	Huynh-Feldt	,935	61,590	,015						
	Límite inferior	,935	17,000	,055						
a. Se ha calculado utilizando alpha =	


2. Exercise_modality


Estimates	
Medida:   MEASURE_1  	
Exercise_modality	Media	Desv. Error	Intervalo de confianza al 95%	
			Límite inferior	Límite superior	
1	1,496	,047	1,397	1,595	
2	2,046	,099	1,839	2,254	


Comparisons in pairs	
Medida:   MEASURE_1  	
(I) Exercise_modality	(J) Exercise_modality	Diferencia de medias (I-J)	Desv. Error	Sig.b	95% de intervalo de confianza para diferenciab	
					Límite inferior	Límite superior	
1	2	-,550*	,104	,000	-,770	-,330	
2	1	,550*	,104	,000	,330	,770	
Se basa en medias marginales estimadas	
*. La diferencia de medias es significativa en el nivel	
b. Ajuste para varias comparaciones: Bonferroni.	


3. Checkpoints

Estimates	
Medida:   MEASURE_1  	
Checkpoints	Media	Desv. Error	Intervalo de confianza al 95%	
			Límite inferior	Límite superior	
1	1,585	,053	1,474	1,696	
2	1,797	,065	1,661	1,933	
3	1,799	,071	1,649	1,948	
4	1,779	,056	1,661	1,897	
5	1,801	,057	1,682	1,921	
6	1,827	,057	1,707	1,946	
7	1,812	,053	1,701	1,923	


Comparisons in pairs	
Medida:   MEASURE_1  	
(I) Checkpoints	(J) Checkpoints	Diferencia de medias (I-J)	Desv. Error	Sig.b	95% de intervalo de confianza para diferenciab	
					Límite inferior	Límite superior	
1	2	-,212*	,025	,000	-,302	-,121	
	3	-,213*	,032	,000	-,326	-,101	
	4	-,193*	,025	,000	-,284	-,103	
	5	-,216*	,021	,000	-,292	-,140	
	6	-,241*	,019	,000	-,310	-,172	
	7	-,227*	,014	,000	-,278	-,175	
2	1	,212*	,025	,000	,121	,302	
	3	-,002	,015	1,000	-,056	,052	
	4	,018	,025	1,000	-,072	,109	
	5	-,004	,024	1,000	-,089	,080	
	6	-,030	,024	1,000	-,114	,054	
	7	-,015	,025	1,000	-,106	,075	
3	1	,213*	,032	,000	,101	,326	
	2	,002	,015	1,000	-,052	,056	
	4	,020	,023	1,000	-,064	,103	
	5	-,003	,024	1,000	-,089	,083	
	6	-,028	,028	1,000	-,128	,073	
	7	-,013	,030	1,000	-,121	,094	
4	1	,193*	,025	,000	,103	,284	
	2	-,018	,025	1,000	-,109	,072	
	3	-,020	,023	1,000	-,103	,064	
	5	-,022	,011	1,000	-,063	,018	
	6	-,048	,028	1,000	-,148	,052	
	7	-,033	,022	1,000	-,113	,046	
5	1	,216*	,021	,000	,140	,292	
	2	,004	,024	1,000	-,080	,089	
	3	,003	,024	1,000	-,083	,089	
	4	,022	,011	1,000	-,018	,063	
	6	-,025	,024	1,000	-,111	,060	
	7	-,011	,017	1,000	-,070	,048	
6	1	,241*	,019	,000	,172	,310	
	2	,030	,024	1,000	-,054	,114	
	3	,028	,028	1,000	-,073	,128	
	4	,048	,028	1,000	-,052	,148	
	5	,025	,024	1,000	-,060	,111	
	7	,014	,013	1,000	-,031	,060	
7	1	,227*	,014	,000	,175	,278	
	2	,015	,025	1,000	-,075	,106	
	3	,013	,030	1,000	-,094	,121	
	4	,033	,022	1,000	-,046	,113	
	5	,011	,017	1,000	-,048	,070	
	6	-,014	,013	1,000	-,060	,031	
Se basa en medias marginales estimadas	
*. La diferencia de medias es significativa en el nivel	
b. Ajuste para varias comparaciones: Bonferroni.	


4. Exercise_modality * Checkpoints	
Medida:   MEASURE_1  	
Exercise_modality	Checkpoints	Media	Desv. Error	Intervalo de confianza al 95%	
				Límite inferior	Límite superior	
1	1	1,354	,050	1,248	1,461	
	2	1,497	,052	1,387	1,607	
	3	1,502	,060	1,375	1,628	
	4	1,500	,047	1,402	1,598	
	5	1,520	,050	1,415	1,625	
	6	1,557	,048	1,455	1,659	
	7	1,544	,045	1,449	1,638	
2	1	1,816	,093	1,619	2,013	
	2	2,097	,115	1,854	2,340	
	3	2,096	,108	1,868	2,323	
	4	2,058	,100	1,847	2,268	
	5	2,082	,100	1,871	2,293	
	6	2,096	,097	1,892	2,300	
	7	2,081	,095	1,881	2,280	


5. Exercise_modality * Checkpoints

Estimates	
Medida:   MEASURE_1  	
Exercise_modality	Checkpoints	Media	Desv. Error	Intervalo de confianza al 95%	
				Límite inferior	Límite superior	
1	1	1,354	,050	1,248	1,461	
	2	1,497	,052	1,387	1,607	
	3	1,502	,060	1,375	1,628	
	4	1,500	,047	1,402	1,598	
	5	1,520	,050	1,415	1,625	
	6	1,557	,048	1,455	1,659	
	7	1,544	,045	1,449	1,638	
2	1	1,816	,093	1,619	2,013	
	2	2,097	,115	1,854	2,340	
	3	2,096	,108	1,868	2,323	
	4	2,058	,100	1,847	2,268	
	5	2,082	,100	1,871	2,293	
	6	2,096	,097	1,892	2,300	
	7	2,081	,095	1,881	2,280	

Comparisons in pairs	
Medida:   MEASURE_1  	
Checkpoints	(I) Exercise_modality	(J) Exercise_modality	Diferencia de medias (I-J)	Desv. Error	Sig.b	95% de intervalo de confianza para diferenciab	
						Límite inferior	Límite superior	
1	1	2	-,462*	,107	,000	-,687	-,236	
	2	1	,462*	,107	,000	,236	,687	
2	1	2	-,600*	,124	,000	-,861	-,340	
	2	1	,600*	,124	,000	,340	,861	
3	1	2	-,594*	,102	,000	-,808	-,380	
	2	1	,594*	,102	,000	,380	,808	
4	1	2	-,558*	,108	,000	-,786	-,329	
	2	1	,558*	,108	,000	,329	,786	
5	1	2	-,562*	,110	,000	-,793	-,330	
	2	1	,562*	,110	,000	,330	,793	
6	1	2	-,539*	,103	,000	-,756	-,322	
	2	1	,539*	,103	,000	,322	,756	
7	1	2	-,537*	,104	,000	-,757	-,317	
	2	1	,537*	,104	,000	,317	,757	
Se basa en medias marginales estimadas	
*. La diferencia de medias es significativa en el nivel	
b. Ajuste para varias comparaciones: Bonferroni.	


6. Exercise_modality * Checkpoints

Estimates	
Medida:   MEASURE_1  	
Exercise_modality	Checkpoints	Media	Desv. Error	Intervalo de confianza al 95%	
				Límite inferior	Límite superior	
1	1	1,354	,050	1,248	1,461	
	2	1,497	,052	1,387	1,607	
	3	1,502	,060	1,375	1,628	
	4	1,500	,047	1,402	1,598	
	5	1,520	,050	1,415	1,625	
	6	1,557	,048	1,455	1,659	
	7	1,544	,045	1,449	1,638	
2	1	1,816	,093	1,619	2,013	
	2	2,097	,115	1,854	2,340	
	3	2,096	,108	1,868	2,323	
	4	2,058	,100	1,847	2,268	
	5	2,082	,100	1,871	2,293	
	6	2,096	,097	1,892	2,300	
	7	2,081	,095	1,881	2,280	


Comparisons in pairs	
Medida:   MEASURE_1  	
Exercise_modality	(I) Checkpoints	(J) Checkpoints	Diferencia de medias (I-J)	Desv. Error	Sig.b	95% de intervalo de confianza para diferenciab	
						Límite inferior	Límite superior	
1	1	2	-,142*	,024	,000	-,228	-,057	
		3	-,147*	,039	,029	-,285	-,009	
		4	-,146*	,033	,007	-,262	-,029	
		5	-,166*	,028	,000	-,266	-,065	
		6	-,203*	,028	,000	-,302	-,104	
		7	-,189*	,032	,000	-,302	-,076	
	2	1	,142*	,024	,000	,057	,228	
		3	-,005	,032	1,000	-,120	,110	
		4	-,003	,028	1,000	-,104	,097	
		5	-,023	,030	1,000	-,130	,083	
		6	-,060	,029	1,000	-,162	,042	
		7	-,047	,033	1,000	-,165	,071	
	3	1	,147*	,039	,029	,009	,285	
		2	,005	,032	1,000	-,110	,120	
		4	,002	,026	1,000	-,091	,094	
		5	-,019	,031	1,000	-,128	,091	
		6	-,055	,030	1,000	-,164	,053	
		7	-,042	,034	1,000	-,163	,080	
	4	1	,146*	,033	,007	,029	,262	
		2	,003	,028	1,000	-,097	,104	
		3	-,002	,026	1,000	-,094	,091	
		5	-,020	,016	1,000	-,078	,037	
		6	-,057	,026	,840	-,149	,034	
		7	-,043	,021	1,000	-,120	,033	
	5	1	,166*	,028	,000	,065	,266	
		2	,023	,030	1,000	-,083	,130	
		3	,019	,031	1,000	-,091	,128	
		4	,020	,016	1,000	-,037	,078	
		6	-,037	,022	1,000	-,117	,043	
		7	-,023	,021	1,000	-,098	,051	
	6	1	,203*	,028	,000	,104	,302	
		2	,060	,029	1,000	-,042	,162	
		3	,055	,030	1,000	-,053	,164	
		4	,057	,026	,840	-,034	,149	
		5	,037	,022	1,000	-,043	,117	
		7	,014	,021	1,000	-,061	,088	
	7	1	,189*	,032	,000	,076	,302	
		2	,047	,033	1,000	-,071	,165	
		3	,042	,034	1,000	-,080	,163	
		4	,043	,021	1,000	-,033	,120	
		5	,023	,021	1,000	-,051	,098	
		6	-,014	,021	1,000	-,088	,061	
2	1	2	-,281*	,044	,000	-,436	-,125	
		3	-,279*	,038	,000	-,414	-,145	
		4	-,241*	,030	,000	-,347	-,136	
		5	-,266*	,026	,000	-,358	-,174	
		6	-,280*	,034	,000	-,400	-,159	
		7	-,264*	,031	,000	-,373	-,155	
	2	1	,281*	,044	,000	,125	,436	
		3	,001	,016	1,000	-,055	,058	
		4	,039	,038	1,000	-,096	,175	
		5	,015	,039	1,000	-,123	,153	
		6	,001	,049	1,000	-,174	,176	
		7	,017	,049	1,000	-,157	,190	
	3	1	,279*	,038	,000	,145	,414	
		2	-,001	,016	1,000	-,058	,055	
		4	,038	,029	1,000	-,067	,143	
		5	,013	,032	1,000	-,101	,128	
		6	,000	,042	1,000	-,149	,148	
		7	,015	,041	1,000	-,132	,162	
	4	1	,241*	,030	,000	,136	,347	
		2	-,039	,038	1,000	-,175	,096	
		3	-,038	,029	1,000	-,143	,067	
		5	-,024	,013	1,000	-,071	,022	
		6	-,038	,042	1,000	-,187	,111	
		7	-,023	,041	1,000	-,170	,124	
	5	1	,266*	,026	,000	,174	,358	
		2	-,015	,039	1,000	-,153	,123	
		3	-,013	,032	1,000	-,128	,101	
		4	,024	,013	1,000	-,022	,071	
		6	-,014	,039	1,000	-,152	,125	
		7	,002	,039	1,000	-,136	,139	
	6	1	,280*	,034	,000	,159	,400	
		2	-,001	,049	1,000	-,176	,174	
		3	,000	,042	1,000	-,148	,149	
		4	,038	,042	1,000	-,111	,187	
		5	,014	,039	1,000	-,125	,152	
		7	,015	,012	1,000	-,028	,058	
	7	1	,264*	,031	,000	,155	,373	
		2	-,017	,049	1,000	-,190	,157	
		3	-,015	,041	1,000	-,162	,132	
		4	,023	,041	1,000	-,124	,170	
		5	-,002	,039	1,000	-,139	,136	
		6	-,015	,012	1,000	-,058	,028	
Se basa en medias marginales estimadas	
*. La diferencia de medias es significativa en el nivel	
b. Ajuste para varias comparaciones: Bonferroni.	


General linear model. Ventilation (VE)


Intra-subject factors	
Medida:   MEASURE_1  	
Exercise_modality	Checkpoints	Variable dependiente	
1	1	HS_VE_P1	
	2	HS_VE_P2	
	3	HS_VE_P3	
	4	HS_VE_P4	
	5	HS_VE_P5	
	6	HS_VE_P6	
	7	HS_VE_P7	
2	1	CYC_VE_P1	
	2	CYC_VE_P2	
	3	CYC_VE_P3	
	4	CYC_VE_P4	
	5	CYC_VE_P5	
	6	CYC_VE_P6	
	7	CYC_VE_P7	

Descriptive statistics	
	Media	Desv. Desviación	N	
HS_VE_P1	37,5130	5,58020	18	
HS_VE_P2	42,5611	5,69981	18	
HS_VE_P3	42,3204	7,16442	18	
HS_VE_P4	43,5444	6,82945	18	
HS_VE_P5	44,1815	6,61718	18	
HS_VE_P6	45,4148	5,83331	18	
HS_VE_P7	45,9981	6,77754	18	
CYC_VE_P1	45,1537	10,57355	18	
CYC_VE_P2	53,0426	13,44260	18	
CYC_VE_P3	54,0722	12,65563	18	
CYC_VE_P4	53,4537	11,34734	18	
CYC_VE_P5	56,9315	13,49191	18	
CYC_VE_P6	54,5759	11,01939	18	
CYC_VE_P7	58,5759	19,38523	18	


Intra-subject factors	
Medida:   MEASURE_1  	
Origen	Tipo III de suma de cuadrados	gl	Media cuadrática	F	Sig.	Eta parcial al cuadrado	Parámetro sin centralidad	Potencia observadaa	
Exercise_modality	Esfericidad asumida	7092,467	1	7092,467	14,087	,002	,453	14,087	,942	
	Greenhouse-Geisser	7092,467	1,000	7092,467	14,087	,002	,453	14,087	,942	
	Huynh-Feldt	7092,467	1,000	7092,467	14,087	,002	,453	14,087	,942	
	Límite inferior	7092,467	1,000	7092,467	14,087	,002	,453	14,087	,942	
Error(Exercise_modality)	Esfericidad asumida	8559,032	17	503,472						
	Greenhouse-Geisser	8559,032	17,000	503,472						
	Huynh-Feldt	8559,032	17,000	503,472						
	Límite inferior	8559,032	17,000	503,472						
Checkpoints	Esfericidad asumida	2615,368	6	435,895	12,678	,000	,427	76,066	1,000	
	Greenhouse-Geisser	2615,368	1,976	1323,828	12,678	,000	,427	25,046	,994	
	Huynh-Feldt	2615,368	2,234	1170,833	12,678	,000	,427	28,319	,997	
	Límite inferior	2615,368	1,000	2615,368	12,678	,002	,427	12,678	,918	
Error(Checkpoints)	Esfericidad asumida	3507,032	102	34,383						
	Greenhouse-Geisser	3507,032	33,585	104,421						
	Huynh-Feldt	3507,032	37,974	92,353						
	Límite inferior	3507,032	17,000	206,296						
Exercise_modality * Checkpoints	Esfericidad asumida	190,610	6	31,768	,884	,510	,049	5,303	,336	
	Greenhouse-Geisser	190,610	1,851	102,962	,884	,416	,049	1,636	,183	
	Huynh-Feldt	190,610	2,068	92,185	,884	,425	,049	1,828	,192	
	Límite inferior	190,610	1,000	190,610	,884	,360	,049	,884	,144	
Error(Exercise_modality*Checkpoints)	Esfericidad asumida	3665,999	102	35,941						
	Greenhouse-Geisser	3665,999	31,472	116,486						
	Huynh-Feldt	3665,999	35,151	104,293						
	Límite inferior	3665,999	17,000	215,647						
a. Se ha calculado utilizando alpha =	


2. Exercise_modality

Estimates	
Medida:   MEASURE_1  	
Exercise_modality	Media	Desv. Error	Intervalo de confianza al 95%	
			Límite inferior	Límite superior	
1	43,076	1,426	40,067	46,085	
2	53,687	2,624	48,150	59,223	


Comparisons in pairs	
Medida:   MEASURE_1  	
(I) Exercise_modality	(J) Exercise_modality	Diferencia de medias (I-J)	Desv. Error	Sig.b	95% de intervalo de confianza para diferenciab	
					Límite inferior	Límite superior	
1	2	-10,610*	2,827	,002	-16,575	-4,646	
2	1	10,610*	2,827	,002	4,646	16,575	
Se basa en medias marginales estimadas	
*. La diferencia de medias es significativa en el nivel	
b. Ajuste para varias comparaciones: Bonferroni.	

3. Checkpoints

Estimates	
Medida:   MEASURE_1  	
Checkpoints	Media	Desv. Error	Intervalo de confianza al 95%	
			Límite inferior	Límite superior	
1	41,333	1,383	38,416	44,250	
2	47,802	1,808	43,987	51,616	
3	48,196	1,912	44,163	52,230	
4	48,499	1,656	45,004	51,994	
5	50,556	1,755	46,854	54,259	
6	49,995	1,489	46,854	53,137	
7	52,287	2,469	47,079	57,495	

Comparisons in pairs	
Medida:   MEASURE_1  	
(I) Checkpoints	(J) Checkpoints	Diferencia de medias (I-J)	Desv. Error	Sig.b	95% de intervalo de confianza para diferenciab	
					Límite inferior	Límite superior	
1	2	-6,469*	,700	,000	-8,963	-3,974	
	3	-6,863*	,848	,000	-9,885	-3,841	
	4	-7,166*	,678	,000	-9,585	-4,747	
	5	-9,223*	1,102	,000	-13,152	-5,294	
	6	-8,662*	,639	,000	-10,942	-6,383	
	7	-10,954*	2,154	,002	-18,634	-3,273	
2	1	6,469*	,700	,000	3,974	8,963	
	3	-,394	,360	1,000	-1,677	,888	
	4	-,697	,535	1,000	-2,603	1,209	
	5	-2,755	1,315	1,000	-7,444	1,934	
	6	-2,194	,921	,613	-5,478	1,091	
	7	-4,485	2,170	1,000	-12,224	3,253	
3	1	6,863*	,848	,000	3,841	9,885	
	2	,394	,360	1,000	-,888	1,677	
	4	-,303	,451	1,000	-1,911	1,305	
	5	-2,360	1,281	1,000	-6,926	2,206	
	6	-1,799	,942	1,000	-5,159	1,561	
	7	-4,091	2,137	1,000	-11,711	3,529	
4	1	7,166*	,678	,000	4,747	9,585	
	2	,697	,535	1,000	-1,209	2,603	
	3	,303	,451	1,000	-1,305	1,911	
	5	-2,057	1,115	1,000	-6,034	1,919	
	6	-1,496	,662	,781	-3,857	,864	
	7	-3,788	2,110	1,000	-11,311	3,735	
5	1	9,223*	1,102	,000	5,294	13,152	
	2	2,755	1,315	1,000	-1,934	7,444	
	3	2,360	1,281	1,000	-2,206	6,926	
	4	2,057	1,115	1,000	-1,919	6,034	
	6	,561	,784	1,000	-2,235	3,357	
	7	-1,731	2,390	1,000	-10,252	6,790	
6	1	8,662*	,639	,000	6,383	10,942	
	2	2,194	,921	,613	-1,091	5,478	
	3	1,799	,942	1,000	-1,561	5,159	
	4	1,496	,662	,781	-,864	3,857	
	5	-,561	,784	1,000	-3,357	2,235	
	7	-2,292	2,172	1,000	-10,036	5,453	
7	1	10,954*	2,154	,002	3,273	18,634	
	2	4,485	2,170	1,000	-3,253	12,224	
	3	4,091	2,137	1,000	-3,529	11,711	
	4	3,788	2,110	1,000	-3,735	11,311	
	5	1,731	2,390	1,000	-6,790	10,252	
	6	2,292	2,172	1,000	-5,453	10,036	
Se basa en medias marginales estimadas	
*. La diferencia de medias es significativa en el nivel	
b. Ajuste para varias comparaciones: Bonferroni.	


4. Exercise_modality * Checkpoints	
Medida:   MEASURE_1  	
Exercise_modality	Checkpoints	Media	Desv. Error	Intervalo de confianza al 95%	
				Límite inferior	Límite superior	
1	1	37,513	1,315	34,738	40,288	
	2	42,561	1,343	39,727	45,396	
	3	42,320	1,689	38,758	45,883	
	4	43,544	1,610	40,148	46,941	
	5	44,181	1,560	40,891	47,472	
	6	45,415	1,375	42,514	48,316	
	7	45,998	1,597	42,628	49,369	
2	1	45,154	2,492	39,896	50,412	
	2	53,043	3,168	46,358	59,727	
	3	54,072	2,983	47,779	60,366	
	4	53,454	2,675	47,811	59,097	
	5	56,931	3,180	50,222	63,641	
	6	54,576	2,597	49,096	60,056	
	7	58,576	4,569	48,936	68,216	


5. Exercise_modality * Checkpoints

Estimates	
Medida:   MEASURE_1  	
Exercise_modality	Checkpoints	Media	Desv. Error	Intervalo de confianza al 95%	
				Límite inferior	Límite superior	
1	1	37,513	1,315	34,738	40,288	
	2	42,561	1,343	39,727	45,396	
	3	42,320	1,689	38,758	45,883	
	4	43,544	1,610	40,148	46,941	
	5	44,181	1,560	40,891	47,472	
	6	45,415	1,375	42,514	48,316	
	7	45,998	1,597	42,628	49,369	
2	1	45,154	2,492	39,896	50,412	
	2	53,043	3,168	46,358	59,727	
	3	54,072	2,983	47,779	60,366	
	4	53,454	2,675	47,811	59,097	
	5	56,931	3,180	50,222	63,641	
	6	54,576	2,597	49,096	60,056	
	7	58,576	4,569	48,936	68,216	


Comparisons in pairs	
Medida:   MEASURE_1  	
Checkpoints	(I) Exercise_modality	(J) Exercise_modality	Diferencia de medias (I-J)	Desv. Error	Sig.b	95% de intervalo de confianza para diferenciab	
						Límite inferior	Límite superior	
1	1	2	-7,641*	2,870	,016	-13,695	-1,586	
	2	1	7,641*	2,870	,016	1,586	13,695	
2	1	2	-10,481*	3,258	,005	-17,355	-3,608	
	2	1	10,481*	3,258	,005	3,608	17,355	
3	1	2	-11,752*	2,980	,001	-18,040	-5,464	
	2	1	11,752*	2,980	,001	5,464	18,040	
4	1	2	-9,909*	2,918	,003	-16,066	-3,753	
	2	1	9,909*	2,918	,003	3,753	16,066	
5	1	2	-12,750*	3,574	,002	-20,290	-5,210	
	2	1	12,750*	3,574	,002	5,210	20,290	
6	1	2	-9,161*	2,899	,006	-15,277	-3,045	
	2	1	9,161*	2,899	,006	3,045	15,277	
7	1	2	-12,578*	4,742	,017	-22,582	-2,574	
	2	1	12,578*	4,742	,017	2,574	22,582	
Se basa en medias marginales estimadas	
*. La diferencia de medias es significativa en el nivel	
b. Ajuste para varias comparaciones: Bonferroni.	


6. Exercise_modality * Checkpoints

Estimates	
Medida:   MEASURE_1  	
Exercise_modality	Checkpoints	Media	Desv. Error	Intervalo de confianza al 95%	
				Límite inferior	Límite superior	
1	1	37,513	1,315	34,738	40,288	
	2	42,561	1,343	39,727	45,396	
	3	42,320	1,689	38,758	45,883	
	4	43,544	1,610	40,148	46,941	
	5	44,181	1,560	40,891	47,472	
	6	45,415	1,375	42,514	48,316	
	7	45,998	1,597	42,628	49,369	
2	1	45,154	2,492	39,896	50,412	
	2	53,043	3,168	46,358	59,727	
	3	54,072	2,983	47,779	60,366	
	4	53,454	2,675	47,811	59,097	
	5	56,931	3,180	50,222	63,641	
	6	54,576	2,597	49,096	60,056	
	7	58,576	4,569	48,936	68,216	


Comparisons in pairs
	
Medida:   MEASURE_1  	
Exercise_modality	(I) Checkpoints	(J) Checkpoints	Diferencia de medias (I-J)	Desv. Error	Sig.b	95% de intervalo de confianza para diferenciab	
						Límite inferior	Límite superior	
1	1	2	-5,048*	,623	,000	-7,271	-2,825	
		3	-4,807*	,935	,002	-8,140	-1,475	
		4	-6,031*	,843	,000	-9,036	-3,026	
		5	-6,669*	,822	,000	-9,601	-3,736	
		6	-7,902*	,723	,000	-10,478	-5,326	
		7	-8,485*	,825	,000	-11,426	-5,544	
	2	1	5,048*	,623	,000	2,825	7,271	
		3	,241	,607	1,000	-1,922	2,404	
		4	-,983	,676	1,000	-3,395	1,428	
		5	-1,620	,867	1,000	-4,711	1,470	
		6	-2,854*	,793	,047	-5,683	-,025	
		7	-3,437*	,841	,016	-6,436	-,438	
	3	1	4,807*	,935	,002	1,475	8,140	
		2	-,241	,607	1,000	-2,404	1,922	
		4	-1,224	,456	,329	-2,850	,402	
		5	-1,861	,725	,420	-4,446	,724	
		6	-3,094	,889	,060	-6,266	,077	
		7	-3,678*	,789	,005	-6,491	-,864	
	4	1	6,031*	,843	,000	3,026	9,036	
		2	,983	,676	1,000	-1,428	3,395	
		3	1,224	,456	,329	-,402	2,850	
		5	-,637	,469	1,000	-2,310	1,036	
		6	-1,870	,820	,750	-4,794	1,053	
		7	-2,454*	,676	,044	-4,865	-,043	
	5	1	6,669*	,822	,000	3,736	9,601	
		2	1,620	,867	1,000	-1,470	4,711	
		3	1,861	,725	,420	-,724	4,446	
		4	,637	,469	1,000	-1,036	2,310	
		6	-1,233	,732	1,000	-3,843	1,377	
		7	-1,817	,571	,114	-3,852	,218	
	6	1	7,902*	,723	,000	5,326	10,478	
		2	2,854*	,793	,047	,025	5,683	
		3	3,094	,889	,060	-,077	6,266	
		4	1,870	,820	,750	-1,053	4,794	
		5	1,233	,732	1,000	-1,377	3,843	
		7	-,583	,425	1,000	-2,099	,932	
	7	1	8,485*	,825	,000	5,544	11,426	
		2	3,437*	,841	,016	,438	6,436	
		3	3,678*	,789	,005	,864	6,491	
		4	2,454*	,676	,044	,043	4,865	
		5	1,817	,571	,114	-,218	3,852	
		6	,583	,425	1,000	-,932	2,099	
2	1	2	-7,889*	1,190	,000	-12,133	-3,645	
		3	-8,919*	1,201	,000	-13,201	-4,636	
		4	-8,300*	,849	,000	-11,326	-5,274	
		5	-11,778*	2,169	,001	-19,512	-4,043	
		6	-9,422*	,930	,000	-12,739	-6,105	
		7	-13,422	4,142	,101	-28,191	1,346	
	2	1	7,889*	1,190	,000	3,645	12,133	
		3	-1,030	,579	1,000	-3,094	1,035	
		4	-,411	1,057	1,000	-4,179	3,357	
		5	-3,889	2,553	1,000	-12,992	5,214	
		6	-1,533	1,459	1,000	-6,734	3,667	
		7	-5,533	4,406	1,000	-21,242	10,175	
	3	1	8,919*	1,201	,000	4,636	13,201	
		2	1,030	,579	1,000	-1,035	3,094	
		4	,619	,858	1,000	-2,441	3,678	
		5	-2,859	2,513	1,000	-11,818	6,100	
		6	-,504	1,433	1,000	-5,613	4,605	
		7	-4,504	4,353	1,000	-20,025	11,018	
	4	1	8,300*	,849	,000	5,274	11,326	
		2	,411	1,057	1,000	-3,357	4,179	
		3	-,619	,858	1,000	-3,678	2,441	
		5	-3,478	2,210	1,000	-11,358	4,403	
		6	-1,122	1,001	1,000	-4,691	2,446	
		7	-5,122	4,367	1,000	-20,691	10,447	
	5	1	11,778*	2,169	,001	4,043	19,512	
		2	3,889	2,553	1,000	-5,214	12,992	
		3	2,859	2,513	1,000	-6,100	11,818	
		4	3,478	2,210	1,000	-4,403	11,358	
		6	2,356	1,480	1,000	-2,921	7,632	
		7	-1,644	4,846	1,000	-18,922	15,634	
	6	1	9,422*	,930	,000	6,105	12,739	
		2	1,533	1,459	1,000	-3,667	6,734	
		3	,504	1,433	1,000	-4,605	5,613	
		4	1,122	1,001	1,000	-2,446	4,691	
		5	-2,356	1,480	1,000	-7,632	2,921	
		7	-4,000	4,276	1,000	-19,247	11,247	
	7	1	13,422	4,142	,101	-1,346	28,191	
		2	5,533	4,406	1,000	-10,175	21,242	
		3	4,504	4,353	1,000	-11,018	20,025	
		4	5,122	4,367	1,000	-10,447	20,691	
		5	1,644	4,846	1,000	-15,634	18,922	
		6	4,000	4,276	1,000	-11,247	19,247	
Se basa en medias marginales estimadas	
*. La diferencia de medias es significativa en el nivel	
b. Ajuste para varias comparaciones: Bonferroni.	


T-Test. VE/VCO2 Slope


Paired sample statistics	
	Media	N	Desv. Desviación	Desv. Error promedio	
Par 1	VE_VCO2_SLOPE_HS	25,6130	18	8,68495	2,04706	
	VE_VCO2_SLOPE_CYC	27,0763	18	7,09272	1,67177	


Paired sample correlations	
	N	Correlación	Sig.	
Par 1	VE_VCO2_SLOPE_HS & VE_VCO2_SLOPE_CYC	18	,422	,081	


Paired sample test	
	Diferencias emparejadas	t	gl	Sig. (bilateral)	
	Media	Desv. Desviación	Desv. Error promedio	95% de intervalo de confianza de la diferencia				
				Inferior	Superior				
Par 1	VE_VCO2_SLOPE_HS - VE_VCO2_SLOPE_CYC	-1,46330	8,58880	2,02440	-5,73441	2,80781	-,723	17	,480	


T-Test. OUES


Paired sample statistics	
	Media	N	Desv. Desviación	Desv. Error promedio	
Par 1	OUES_SLOPE_HS	2,5077	18	,60911	,14357	
	OUES_SLOPE_CYC	2,5623	18	1,30551	,30771	


Paired sample correlations	
	N	Correlación	Sig.	
Par 1	OUES_SLOPE_HS & OUES_SLOPE_CYC	18	,356	,147	


Paired sample test	
	Diferencias emparejadas	t	gl	Sig. (bilateral)	
	Media	Desv. Desviación	Desv. Error promedio	95% de intervalo de confianza de la diferencia				
				Inferior	Superior				
Par 1	OUES_SLOPE_HS - OUES_SLOPE_CYC	-,05452	1,22855	,28957	-,66546	,55642	-,188	17	,853	


General linear model (Lactate)


Intra-subject factors	
Medida:   MEASURE_1  	
Exercise_mode	Checkpoints8	Variable dependiente	
1	1	AcLCT_cte_RT_p0	
	2	AcLCT_cte_RT_p1	
	3	AcLCT_cte_RT_p2	
	4	AcLCT_cte_RT_p3	
	5	AcLCT_cte_RT_p4	
	6	AcLCT_cte_RT_p5	
	7	AcLCT_cte_RT_p6	
	8	AcLCT_cte_RT_p7	
2	1	AcLCT_cte_BICI_p0	
	2	AcLCT_cte_BICI_p1	
	3	AcLCT_cte_BICI_p2	
	4	AcLCT_cte_BICI_p3	
	5	AcLCT_cte_BICI_p4	
	6	AcLCT_cte_BICI_p5	
	7	AcLCT_cte_BICI_p6	
	8	AcLCT_cte_BICI_p7	


Descriptive statistics	
	Media	Desv. Desviación	N	
AcLCT_cte_RT_p0	1,2611	,29132	18	
AcLCT_cte_RT_p1	2,7833	,52720	18	
AcLCT_cte_RT_p2	2,8944	,59059	18	
AcLCT_cte_RT_p3	2,9944	,78100	18	
AcLCT_cte_RT_p4	3,0111	,72994	18	
AcLCT_cte_RT_p5	3,1722	,67459	18	
AcLCT_cte_RT_p6	3,3222	,58867	18	
AcLCT_cte_RT_p7	3,2611	,70138	18	
AcLCT_cte_BICI_p0	1,3500	,34171	18	
AcLCT_cte_BICI_p1	2,8778	,85924	18	
AcLCT_cte_BICI_p2	2,9722	,99043	18	
AcLCT_cte_BICI_p3	2,7722	,94546	18	
AcLCT_cte_BICI_p4	2,7222	,89938	18	
AcLCT_cte_BICI_p5	2,6222	,85444	18	
AcLCT_cte_BICI_p6	2,6611	,97868	18	
AcLCT_cte_BICI_p7	2,3833	,78384	18	


Intra-subject factors	
Medida:   MEASURE_1  	
Origen	Tipo III de suma de cuadrados	gl	Media cuadrática	F	Sig.	Eta parcial al cuadrado	Parámetro sin centralidad	Potencia observadaa	
Exercise_mode	Esfericidad asumida	6,154	1	6,154	2,301	,148	,119	2,301	,299	
	Greenhouse-Geisser	6,154	1,000	6,154	2,301	,148	,119	2,301	,299	
	Huynh-Feldt	6,154	1,000	6,154	2,301	,148	,119	2,301	,299	
	Límite inferior	6,154	1,000	6,154	2,301	,148	,119	2,301	,299	
Error(Exercise_mode)	Esfericidad asumida	45,466	17	2,674						
	Greenhouse-Geisser	45,466	17,000	2,674						
	Huynh-Feldt	45,466	17,000	2,674						
	Límite inferior	45,466	17,000	2,674						
Checkpoints8	Esfericidad asumida	79,764	7	11,395	55,852	,000	,767	390,961	1,000	
	Greenhouse-Geisser	79,764	3,455	23,089	55,852	,000	,767	192,946	1,000	
	Huynh-Feldt	79,764	4,443	17,952	55,852	,000	,767	248,153	1,000	
	Límite inferior	79,764	1,000	79,764	55,852	,000	,767	55,852	1,000	
Error(Checkpoints8)	Esfericidad asumida	24,278	119	,204						
	Greenhouse-Geisser	24,278	58,729	,413						
	Huynh-Feldt	24,278	75,532	,321						
	Límite inferior	24,278	17,000	1,428						
Exercise_mode * Checkpoints8	Esfericidad asumida	8,838	7	1,263	6,928	,000	,290	48,495	1,000	
	Greenhouse-Geisser	8,838	3,329	2,655	6,928	,000	,290	23,064	,980	
	Huynh-Feldt	8,838	4,237	2,086	6,928	,000	,290	29,353	,994	
	Límite inferior	8,838	1,000	8,838	6,928	,017	,290	6,928	,699	
Error(Exercise_mode*Checkpoints8)	Esfericidad asumida	21,687	119	,182						
	Greenhouse-Geisser	21,687	56,595	,383						
	Huynh-Feldt	21,687	72,029	,301						
	Límite inferior	21,687	17,000	1,276						
a. Se ha calculado utilizando alpha =	


2. Exercise_mode

Estimates	
Medida:   MEASURE_1  	
Exercise_mode	Media	Desv. Error	Intervalo de confianza al 95%	
			Límite inferior	Límite superior	
1	2,838	,122	2,581	3,094	
2	2,545	,170	2,187	2,903	


Comparisons in pairs	
Medida:   MEASURE_1  	
(I) Exercise_mode	(J) Exercise_mode	Diferencia de medias (I-J)	Desv. Error	Sig.a	95% de intervalo de confianza para diferenciaa	
					Límite inferior	Límite superior	
1	2	,292	,193	,148	-,114	,699	
2	1	-,292	,193	,148	-,699	,114	
Se basa en medias marginales estimadas	
a. Ajuste para varias comparaciones: Bonferroni.	

3. Checkpoints8

Estimates	
Medida:   MEASURE_1  	
Checkpoints8	Media	Desv. Error	Intervalo de confianza al 95%	
			Límite inferior	Límite superior	
1	1,306	,065	1,169	1,442	
2	2,831	,127	2,562	3,099	
3	2,933	,143	2,631	3,236	
4	2,883	,156	2,554	3,213	
5	2,867	,147	2,557	3,177	
6	2,897	,143	2,596	3,199	
7	2,992	,135	2,706	3,277	
8	2,822	,120	2,570	3,074	


Comparisons in pairs	
Medida:   MEASURE_1  	
(I) Checkpoints8	(J) Checkpoints8	Diferencia de medias (I-J)	Desv. Error	Sig.b	95% de intervalo de confianza para diferenciab	
					Límite inferior	Límite superior	
1	2	-1,525*	,142	,000	-2,049	-1,001	
	3	-1,628*	,150	,000	-2,183	-1,073	
	4	-1,578*	,162	,000	-2,176	-,980	
	5	-1,561*	,158	,000	-2,144	-,978	
	6	-1,592*	,151	,000	-2,151	-1,033	
	7	-1,686*	,149	,000	-2,237	-1,135	
	8	-1,517*	,135	,000	-2,017	-1,016	
2	1	1,525*	,142	,000	1,001	2,049	
	3	-,103	,081	1,000	-,403	,197	
	4	-,053	,101	1,000	-,425	,320	
	5	-,036	,081	1,000	-,334	,262	
	6	-,067	,095	1,000	-,416	,283	
	7	-,161	,105	1,000	-,550	,228	
	8	,008	,094	1,000	-,338	,355	
3	1	1,628*	,150	,000	1,073	2,183	
	2	,103	,081	1,000	-,197	,403	
	4	,050	,066	1,000	-,194	,294	
	5	,067	,078	1,000	-,221	,354	
	6	,036	,079	1,000	-,254	,327	
	7	-,058	,104	1,000	-,444	,328	
	8	,111	,104	1,000	-,273	,495	
4	1	1,578*	,162	,000	,980	2,176	
	2	,053	,101	1,000	-,320	,425	
	3	-,050	,066	1,000	-,294	,194	
	5	,017	,076	1,000	-,263	,296	
	6	-,014	,078	1,000	-,303	,276	
	7	-,108	,107	1,000	-,504	,287	
	8	,061	,103	1,000	-,319	,441	
5	1	1,561*	,158	,000	,978	2,144	
	2	,036	,081	1,000	-,262	,334	
	3	-,067	,078	1,000	-,354	,221	
	4	-,017	,076	1,000	-,296	,263	
	6	-,031	,076	1,000	-,310	,249	
	7	-,125	,101	1,000	-,497	,247	
	8	,044	,095	1,000	-,306	,395	
6	1	1,592*	,151	,000	1,033	2,151	
	2	,067	,095	1,000	-,283	,416	
	3	-,036	,079	1,000	-,327	,254	
	4	,014	,078	1,000	-,276	,303	
	5	,031	,076	1,000	-,249	,310	
	7	-,094	,068	1,000	-,348	,159	
	8	,075	,073	1,000	-,193	,343	
7	1	1,686*	,149	,000	1,135	2,237	
	2	,161	,105	1,000	-,228	,550	
	3	,058	,104	1,000	-,328	,444	
	4	,108	,107	1,000	-,287	,504	
	5	,125	,101	1,000	-,247	,497	
	6	,094	,068	1,000	-,159	,348	
	8	,169*	,039	,012	,025	,314	
8	1	1,517*	,135	,000	1,016	2,017	
	2	-,008	,094	1,000	-,355	,338	
	3	-,111	,104	1,000	-,495	,273	
	4	-,061	,103	1,000	-,441	,319	
	5	-,044	,095	1,000	-,395	,306	
	6	-,075	,073	1,000	-,343	,193	
	7	-,169*	,039	,012	-,314	-,025	
Se basa en medias marginales estimadas	
*. La diferencia de medias es significativa en el nivel	
b. Ajuste para varias comparaciones: Bonferroni.	

4. Exercise_mode * Checkpoints8	
Medida:   MEASURE_1  	
Exercise_mode	Checkpoints8	Media	Desv. Error	Intervalo de confianza al 95%	
				Límite inferior	Límite superior	
1	1	1,261	,069	1,116	1,406	
	2	2,783	,124	2,521	3,046	
	3	2,894	,139	2,601	3,188	
	4	2,994	,184	2,606	3,383	
	5	3,011	,172	2,648	3,374	
	6	3,172	,159	2,837	3,508	
	7	3,322	,139	3,029	3,615	
	8	3,261	,165	2,912	3,610	
2	1	1,350	,081	1,180	1,520	
	2	2,878	,203	2,450	3,305	
	3	2,972	,233	2,480	3,465	
	4	2,772	,223	2,302	3,242	
	5	2,722	,212	2,275	3,169	
	6	2,622	,201	2,197	3,047	
	7	2,661	,231	2,174	3,148	
	8	2,383	,185	1,994	2,773	


5. Exercise_mode * Checkpoints8

Estimates	
Medida:   MEASURE_1  	
Exercise_mode	Checkpoints8	Media	Desv. Error	Intervalo de confianza al 95%	
				Límite inferior	Límite superior	
1	1	1,261	,069	1,116	1,406	
	2	2,783	,124	2,521	3,046	
	3	2,894	,139	2,601	3,188	
	4	2,994	,184	2,606	3,383	
	5	3,011	,172	2,648	3,374	
	6	3,172	,159	2,837	3,508	
	7	3,322	,139	3,029	3,615	
	8	3,261	,165	2,912	3,610	
2	1	1,350	,081	1,180	1,520	
	2	2,878	,203	2,450	3,305	
	3	2,972	,233	2,480	3,465	
	4	2,772	,223	2,302	3,242	
	5	2,722	,212	2,275	3,169	
	6	2,622	,201	2,197	3,047	
	7	2,661	,231	2,174	3,148	
	8	2,383	,185	1,994	2,773	


Comparisons in pairs	
Medida:   MEASURE_1  	
Checkpoints8	(I) Exercise_mode	(J) Exercise_mode	Diferencia de medias (I-J)	Desv. Error	Sig.b	95% de intervalo de confianza para diferenciab	
						Límite inferior	Límite superior	
1	1	2	-,089	,075	,252	-,247	,069	
	2	1	,089	,075	,252	-,069	,247	
2	1	2	-,094	,219	,672	-,557	,368	
	2	1	,094	,219	,672	-,368	,557	
3	1	2	-,078	,256	,765	-,618	,462	
	2	1	,078	,256	,765	-,462	,618	
4	1	2	,222	,264	,411	-,334	,778	
	2	1	-,222	,264	,411	-,778	,334	
5	1	2	,289	,250	,264	-,239	,817	
	2	1	-,289	,250	,264	-,817	,239	
6	1	2	,550*	,223	,025	,079	1,021	
	2	1	-,550*	,223	,025	-1,021	-,079	
7	1	2	,661*	,268	,025	,096	1,227	
	2	1	-,661*	,268	,025	-1,227	-,096	
8	1	2	,878*	,256	,003	,337	1,419	
	2	1	-,878*	,256	,003	-1,419	-,337	
Se basa en medias marginales estimadas	
*. La diferencia de medias es significativa en el nivel	
b. Ajuste para varias comparaciones: Bonferroni.	


6. Exercise_mode * Checkpoints8

Estimates	
Medida:   MEASURE_1  	
Exercise_mode	Checkpoints8	Media	Desv. Error	Intervalo de confianza al 95%	
				Límite inferior	Límite superior	
1	1	1,261	,069	1,116	1,406	
	2	2,783	,124	2,521	3,046	
	3	2,894	,139	2,601	3,188	
	4	2,994	,184	2,606	3,383	
	5	3,011	,172	2,648	3,374	
	6	3,172	,159	2,837	3,508	
	7	3,322	,139	3,029	3,615	
	8	3,261	,165	2,912	3,610	
2	1	1,350	,081	1,180	1,520	
	2	2,878	,203	2,450	3,305	
	3	2,972	,233	2,480	3,465	
	4	2,772	,223	2,302	3,242	
	5	2,722	,212	2,275	3,169	
	6	2,622	,201	2,197	3,047	
	7	2,661	,231	2,174	3,148	
	8	2,383	,185	1,994	2,773	


Comparisons in pairs	
Medida:   MEASURE_1  	
Exercise_mode	(I) Checkpoints8	(J) Checkpoints8	Diferencia de medias (I-J)	Desv. Error	Sig.b	95% de intervalo de confianza para diferenciab	
						Límite inferior	Límite superior	
1	1	2	-1,522*	,125	,000	-1,986	-1,059	
		3	-1,633*	,129	,000	-2,111	-1,156	
		4	-1,733*	,177	,000	-2,387	-1,080	
		5	-1,750*	,161	,000	-2,346	-1,154	
		6	-1,911*	,153	,000	-2,477	-1,345	
		7	-2,061*	,144	,000	-2,593	-1,530	
		8	-2,000*	,181	,000	-2,668	-1,332	
	2	1	1,522*	,125	,000	1,059	1,986	
		3	-,111	,090	1,000	-,444	,222	
		4	-,211	,162	1,000	-,810	,387	
		5	-,228	,128	1,000	-,699	,244	
		6	-,389	,147	,469	-,931	,153	
		7	-,539*	,135	,026	-1,037	-,041	
		8	-,478	,154	,182	-1,048	,092	
	3	1	1,633*	,129	,000	1,156	2,111	
		2	,111	,090	1,000	-,222	,444	
		4	-,100	,089	1,000	-,430	,230	
		5	-,117	,068	1,000	-,367	,134	
		6	-,278	,094	,245	-,625	,069	
		7	-,428*	,103	,018	-,807	-,049	
		8	-,367	,119	,191	-,807	,074	
	4	1	1,733*	,177	,000	1,080	2,387	
		2	,211	,162	1,000	-,387	,810	
		3	,100	,089	1,000	-,230	,430	
		5	-,017	,060	1,000	-,237	,204	
		6	-,178	,100	1,000	-,547	,191	
		7	-,328	,145	1,000	-,863	,208	
		8	-,267	,143	1,000	-,797	,264	
	5	1	1,750*	,161	,000	1,154	2,346	
		2	,228	,128	1,000	-,244	,699	
		3	,117	,068	1,000	-,134	,367	
		4	,017	,060	1,000	-,204	,237	
		6	-,161	,089	1,000	-,492	,169	
		7	-,311	,130	,786	-,790	,168	
		8	-,250	,138	1,000	-,759	,259	
	6	1	1,911*	,153	,000	1,345	2,477	
		2	,389	,147	,469	-,153	,931	
		3	,278	,094	,245	-,069	,625	
		4	,178	,100	1,000	-,191	,547	
		5	,161	,089	1,000	-,169	,492	
		7	-,150	,102	1,000	-,526	,226	
		8	-,089	,107	1,000	-,483	,305	
	7	1	2,061*	,144	,000	1,530	2,593	
		2	,539*	,135	,026	,041	1,037	
		3	,428*	,103	,018	,049	,807	
		4	,328	,145	1,000	-,208	,863	
		5	,311	,130	,786	-,168	,790	
		6	,150	,102	1,000	-,226	,526	
		8	,061	,059	1,000	-,157	,279	
	8	1	2,000*	,181	,000	1,332	2,668	
		2	,478	,154	,182	-,092	1,048	
		3	,367	,119	,191	-,074	,807	
		4	,267	,143	1,000	-,264	,797	
		5	,250	,138	1,000	-,259	,759	
		6	,089	,107	1,000	-,305	,483	
		7	-,061	,059	1,000	-,279	,157	
2	1	2	-1,528*	,233	,000	-2,390	-,666	
		3	-1,622*	,265	,000	-2,600	-,644	
		4	-1,422*	,256	,001	-2,369	-,475	
		5	-1,372*	,249	,001	-2,293	-,452	
		6	-1,272*	,237	,001	-2,147	-,397	
		7	-1,311*	,267	,004	-2,299	-,324	
		8	-1,033*	,217	,005	-1,836	-,231	
	2	1	1,528*	,233	,000	,666	2,390	
		3	-,094	,097	1,000	-,454	,265	
		4	,106	,096	1,000	-,251	,462	
		5	,156	,072	1,000	-,112	,423	
		6	,256	,110	,932	-,152	,664	
		7	,217	,155	1,000	-,358	,791	
		8	,494*	,125	,028	,033	,956	
	3	1	1,622*	,265	,000	,644	2,600	
		2	,094	,097	1,000	-,265	,454	
		4	,200	,105	1,000	-,187	,587	
		5	,250	,128	1,000	-,225	,725	
		6	,350	,144	,729	-,181	,881	
		7	,311	,181	1,000	-,359	,982	
		8	,589	,171	,089	-,045	1,223	
	4	1	1,422*	,256	,001	,475	2,369	
		2	-,106	,096	1,000	-,462	,251	
		3	-,200	,105	1,000	-,587	,187	
		5	,050	,127	1,000	-,420	,520	
		6	,150	,102	1,000	-,226	,526	
		7	,111	,140	1,000	-,407	,630	
		8	,389	,120	,132	-,053	,831	
	5	1	1,372*	,249	,001	,452	2,293	
		2	-,156	,072	1,000	-,423	,112	
		3	-,250	,128	1,000	-,725	,225	
		4	-,050	,127	1,000	-,520	,420	
		6	,100	,127	1,000	-,368	,568	
		7	,061	,151	1,000	-,497	,619	
		8	,339	,124	,406	-,121	,799	
	6	1	1,272*	,237	,001	,397	2,147	
		2	-,256	,110	,932	-,664	,152	
		3	-,350	,144	,729	-,881	,181	
		4	-,150	,102	1,000	-,526	,226	
		5	-,100	,127	1,000	-,568	,368	
		7	-,039	,089	1,000	-,368	,290	
		8	,239	,089	,439	-,090	,568	
	7	1	1,311*	,267	,004	,324	2,299	
		2	-,217	,155	1,000	-,791	,358	
		3	-,311	,181	1,000	-,982	,359	
		4	-,111	,140	1,000	-,630	,407	
		5	-,061	,151	1,000	-,619	,497	
		6	,039	,089	1,000	-,290	,368	
		8	,278	,091	,206	-,060	,615	
	8	1	1,033*	,217	,005	,231	1,836	
		2	-,494*	,125	,028	-,956	-,033	
		3	-,589	,171	,089	-1,223	,045	
		4	-,389	,120	,132	-,831	,053	
		5	-,339	,124	,406	-,799	,121	
		6	-,239	,089	,439	-,568	,090	
		7	-,278	,091	,206	-,615	,060	
Se basa en medias marginales estimadas	
*. La diferencia de medias es significativa en el nivel	
b. Ajuste para varias comparaciones: Bonferroni.	
